# FinTextSim: a domain-specific sentence-transformer for extracting predictive latent topics from financial disclosures

**DOI:** 10.3389/frai.2026.1752103

**Published:** 2026-03-02

**Authors:** Simon Jehnen, Javier Villalba-Díez, Joaquín Ordieres-Meré

**Affiliations:** 1 DEGIN Doctoral Program, Department of Industrial Management, Escuela Técnica Superior de Ingenieros Industriales, Universidad Politécnica de Madrid, Madrid, Spain; 2 Beta Klinik GmbH, Bonn, Germany; 3 Fakultät für Wirtschaft, Hochschule Heilbronn, Heilbronn, Germany; 4 Department of Mechanical Engineering, Universidad de La Rioja, Logroño, Spain

**Keywords:** artificial intelligence, BERTopic, company performance prediction, FinTextSim, LDA, machine learning, topic modeling

## Abstract

Recent advancements in information availability and computational capabilities have transformed the analysis of annual reports, integrating traditional financial metrics with insights from textual data. To extract actionable insights from this wealth of textual data, automated review processes, such as topic modeling, are essential. This study benchmarks classical approaches against contemporary neural techniques and introduces FinTextSim, a sentence-transformer finetuned for financial text. Using Item 7 and Item 7A of 10-K filings from S&P 500 companies (2016–2023), we systematically evaluate these models qualitatively and quantitatively. BERTopic in combination with FinTextSim consistently outperforms all alternatives, producing notably clearer, more coherent and financially relevant topic clusters. Compared to the most widely used standard embedding models and financial baselines, FinTextSim improves intratopic similarity by up to 71% and reduces intertopic similarity by more than 108%, highlighting the importance of domain-specific embeddings. Crucially, these qualitative gains translate into quantitative predictive benefits: incorporating FinTextSim-derived topic features into a logistic regression framework for corporate performance prediction leads to a statistically significant two-percentage-point increase in both ROC-AUC and F1-score over a purely financial baseline. In contrast, off-the-shelf sentence-transformers and classical topic models introduce noise that degrades predictive performance. For non-linear classifiers, several textual representations yield modest gains, reflecting their greater capacity to absorb noisier features. However, FinTextSim remains the most stable and consistently strong performer across both linear and non-linear settings. Overall, FinTextSim acts as a domain-adapted information filter, translating unstructured financial text into structured, semantically rich representations that human analysts and generic models often overlook. By bridging interpretability and predictive utility, it enables the extraction of economically relevant information from corporate narratives and supports more effective decision-making, resource allocation, and corporate performance forecasting.

## 1 Introduction

In recent years, the increasing availability of information (Chen and Ji, 2025; Sun et al., 2026) and advances in computational capabilities have transformed the analysis of annual reports, including 10-K filings. These filings are among the most critical disclosures (Griffin, 2003; Hajek and Munk, 2024), providing a standardized snapshot of a company's financial situation through both numerical and textual data (Masson and Paroubek, 2020). Traditional evaluations of 10-K filings have focused on retrospective quantitative financial metrics, while textual data remains underexplored (Hida and Do Nascimento, 2026). However, growing evidence shows that qualitative textual components also carry predictive power for future performance (Cohen et al., 2020; Ashtiani and Raahemi, 2023; Nazareth and Reddy, 2023; Zhu, 2026; Wang et al., 2023; Frankel et al., 2022; Siano, 2025). While these studies demonstrate the predictive potential of textual disclosures, they largely adopt end-to-end predictive frameworks and provide limited insight into how alternative textual representations, particularly topic-based representations, differ in their ability to extract economically meaningful information. Thus, integrating these textual insights with financial metrics provides a more comprehensive basis for decision-making, benefiting investors, analysts, and regulators (Hsieh and Hristova, 2022; Ueda et al., 2024).

Within 10-K filings, Item 7 and Item 7A are particularly valuable. Item 7, the Management Discussion & Analysis (MD&A), presents management's perspective on various aspects, including operations, performance, risks, opportunities, and strategies to address future challenges (Cohen et al., 2020). Item 7A provides qualitative and quantitative disclosures about market risk. As 10-K filings are mandatory for publicly traded companies, they represent a rich source of financial text that requires systematic and scalable analysis. Manual review, however, is both time-consuming and prone to subjectivity bias (Hagen, 2018; Huang et al., 2025). The growing volume of available information (Rashid et al., 2019; Wang Y. et al., 2024) further increases risk of information overload (Lu, 2022), making it essential to allocate resources efficiently (Liu, 2022; Pufahl et al., 2025). Automated approaches, such as topic modeling, address these challenges by uncovering latent topics and summarizing large text corpora (Blei et al., 2003; Song et al., 2025; Curiskis et al., 2020). A key advantage of topic modeling is its unsupervised nature. While supervised approaches often require extensive annotated datasets, which are infeasible in most real-world settings, unsupervised methods scale more efficiently (Taha, 2023).

Classical topic modeling approaches, including Latent Dirichlet Allocation (LDA) and Non-negative Matrix Factorization (NMF), rely on the bag-of-words (BoW) assumption. This assumption posits that each document is treated as a collection of words, disregarding their sequential order. However, this limits the model's ability to capture the semantic meaning of text. Neural topic modeling approaches address this issue by employing contextual embeddings (Blair et al., 2020), which capture semantic and contextual relationships between texts (Booker et al., 2024; Bhattacharya and Mickovic, 2024). Sentence-transformers further improve efficiency and semantic similarity comparisons (Reimers and Gurevych, 2019). These text representations are crucial, as they must faithfully reflect a document's content while distinguishing it from others (Sun et al., 2026), enabling advanced applications such as BERTopic (Grootendorst, 2022). Despite the widespread use of topic modeling and contextual embeddings in general Natural Language Processing (NLP), little is known about their effectiveness in financial applications, where specialized terminology and domain-specific context are critical (Bhattacharya and Mickovic, 2024).

To address this gap, we develop and evaluate FinTextSim, a sentence-transformer finetuned specifically for financial text. General-purpose models, such as all-MiniLM-L6-v2 (AM) and all-mpnet-base-v2 (MPNET), have become standard baselines due to their strong performance across a wide range of domains. Yet, they are not optimized for the semantic and contextual nuances of financial language. Furthermore, existing models tailored for the financial domain are primarily optimized for sentiment analysis (e.g., Araci, 2019; Li et al., 2023; Guo et al., 2024). As a result, their suitability for topic modeling and semantic clustering in financial text remains an open empirical question. In contrast, FinTextSim is explicitly designed to capture domain-specific semantic structure. Functioning as a domain-adapted information filter, FinTextSim mitigates a fundamental information processing and retrieval bottleneck in financial text analysis. By distilling unstructured narratives into structured, semantically rich representations that emphasize economically meaningful relations, it extracts signals often overlooked by both human analysts and generic models. Beyond model development, we systematically evaluate multiple topic modeling algorithms, comparing classical approaches with contemporary neural techniques. This dual benchmarking across embedding models and topic modeling paradigms provides the first comprehensive evaluation of topic modeling for financial text. Moreover, we demonstrate the practical relevance of FinTextSim-enhanced BERTopic, which generates higher-quality and financially relevant insights with direct implications for research, business valuation, and stock price prediction.

Extending this analysis, we integrate the outputs of topic models into a machine learning (ML) framework to assess their informational value for corporate performance prediction. Corporate performance prediction is a central objective in accounting and financial research, as accurate forecasts are closely linked to future excess investment returns (Veganzones and Severin, 2025; Cao and You, 2024; Easton et al., 2024; Uddin et al., 2022; Chen et al., 2022). Although several studies emphasize the potential of NLP and topic modeling to enhance corporate performance prediction (Peng, 2025; Hajek and Munk, 2024; Theodorakopoulos et al., 2025; Lee and Anderl, 2025), systematic evidence on how alternative textual representations, particularly topic-based representations, contribute incremental value when combined with quantitative financial indicators remains limited. To address this second gap, our approach combines topic-document distributions derived from topic models with fundamental financial indicators, allowing ML models to exploit both quantitative and qualitative information. This design enables us to assess which topic modeling approach most effectively quantifies qualitative textual information to improve corporate performance prediction, and to evaluate the robustness of these textual representations across both linear and non-linear predictive frameworks.

We will explore the following research questions based on Item 7 and Item 7A from S&P500 companies between 2016 and 2023:

RQ1 How can we leverage contextual embeddings for the financial domain?
RQ2 Which topic modeling approach provides the most qualitative and coherent topics?
RQ3 Which topic modeling approach proves best in organizing and summarizing our large-scale financial text dataset?
RQ4 Does topic modeling improve corporate performance prediction?

The rest of the paper hereinafter is organized as follows. Section 2 reviews the state-of-the-art literature and methodologies. Section 3 describes our study's materials and methods, including the training procedure of FinTextSim. Section 4 presents and discusses the main findings. Finally, Section 5 provides the conclusion. This structure ensures a clear and logical progression, enabling a thorough understanding of our study's contributions.

## 2 State of the art

The following subsections provide an overview of topic modeling approaches and corporate performance prediction. They will set the foundation for understanding the algorithms and methodologies.

### 2.1 Classical topic modeling approaches

Among classical topic modeling approaches, we highlight LDA and NMF. Both operate under the BoW assumption, treating each document as a mixture of underlying topics and each topic as a mixture of words. Accordingly, they assign prevalence of terms to topics (β) and topics to documents (γ) (Blei et al., 2003). To ensure robust performance, several preprocessing steps are typically applied, including tokenization, stopword removal and lemmatization or stemming of words (Bellstam et al., 2021; Fu et al., 2021; Albalawi et al., 2020).

#### 2.1.1 Latent dirichlet allocation

LDA is the most widely applied topic modeling approach in literature. It is a three-level parametric hierarchical Bayesian model. By defining a hypothetical generative process for documents, LDA works backwards to infer the topics that could have generated the documents (Abdelrazek et al., 2023). The model is governed by three key hyperparameters (Blei et al., 2003): the number of topics (*k*), the concentration parameter of the Dirichlet prior of the document-topic distribution (α), and the parameter controlling the distribution of words across topics (η) (Fernandes et al., 2020). These hyperparameters significantly influence the quality and stability of the generated topics. Yet, their selection remains challenging due to the inherent complexity of textual data (Maier et al., 2018; Agrawal et al., 2018).

Despite its popularity, LDA faces several limitations. LDA is sensitive to the order of training data. As a result, topic structures can vary when the training data is shuffled, introducing systematic errors into studies (Agrawal et al., 2018). Furthermore, overlapping topics can occur as LDA extracts topics from word distributions independently Campbell et al., (2015).

LDA has been used in various fields. Bao and Datta (2014) pioneered the integration of unsupervised learning methods into Management Accounting and Finance using LDA to analyze risk disclosures from 10-K reports. Dyer et al. (2017) examined topics contributing to the lengthening of 10-K reports over time, while Brown et al. (2020) identified topics predicting financial misreporting. Deveikyte et al. (2022) employ LDA to predict market volatility. In additional financial studies, LDA has been used to quantify the economic content in communications, identify central subjects or to estimate innovation capabilities, among other applications (Jegadeesh and Wu, 2017; Lowry et al., 2020; Bellstam et al., 2021; García-Méndez et al., 2023).

#### 2.1.2 Non-negative matrix factorization

NMF takes a decompositional, non-probabilistic approach to topic modeling, factorizing the input document-term-matrix *A* into the product of term-topic-matrix *W* and topic-document-matrix *H* (Lee and Seung, 1999). By evaluating the discrepancy between *A* and *W*×*H* using the squared Frobenius norm, the topic modeling problem is framed as an optimization task restricted to non-negative entries (Wang and Zhang, 2023). Unlike LDA, NMF does not rely on Bayesian priors, although the number of topics still needs to be specified by the user.

While NMF offers advantages in simplicity and computational efficiency (Egger and Yu, 2022), it also faces several challenges. Compared to LDA, it lacks a solid statistical foundation and a defined generative model. Additionally, NMF relies on anchor words to enforce a block diagonal structure in the term-topic matrix *W*, ensuring consistent solutions (Donoho and Stodden, 2003; Gillis and Vavasis, 2014). This assumption posits that each topic is associated with a unique anchor word, absent in other topics (Gillis and Vavasis, 2014). Given the multifaceted nature of words, this assumption can be considered as fragile (Wang and Zhang, 2023). Another assumption of NMF is that each topic has at least one “pure document,” a document discussing only that specific topic Gillis and Vavasis, (2014). This assumption is particularly fragile for longer documents.

NMF has applications in various fields and domains. In finance, Chen et al. (2017) used NMF and other topic modeling methods on 10-K and 8-K filings of bank holding companies to distinguish failed from non-failed banks. Additionally, Cai et al. (2022) applied NMF to assess the impact of risk factor disclosures on bond pricing. In other fields, NMF has been primarily employed for short-text topic modeling (Chen et al., 2019; Albalawi et al., 2020; Egger and Yu, 2022).

#### 2.1.3 Wrapup of classical topic modeling approaches

Classical topic modeling approaches offer both, advantages and disadvantages. A main advantage is the easier interpretation of hyperparameters, aiding in troubleshooting and model interpretation. However, disadvantages become increasingly pronounced with more complex corpora (Abdelrazek et al., 2023). Classical models are particularly susceptible to the following issues:

1. BoW Assumption: context and semantic relationships cannot be captured (Murphy et al., 2024); misrepresentation of topics and documents possible (Grootendorst, 2022),
2. Interpretability of topics (Campbell et al., 2015; Song et al., 2025),
3. Reliability, validity, and subjectivity: outcomes depend heavily on manual preprocessing choices and hyperparameter selection (Baden et al., 2022).

### 2.2 Contemporary topic modeling approaches

Modern methodologies address the issues of classical topic modeling approaches by utilizing advanced text embedding techniques (Blair et al., 2020). The following subsections provide an overview of the evolution of contemporary techniques and a detailed examination of BERTopic, a state-of-the-art topic modeling approach.

#### 2.2.1 Evolution of contemporary topic modeling approaches

The integration of contextual embeddings has transformed topic modeling by moving beyond the BoW assumption, enabling better capturing of semantic relationships within text (Blair et al., 2020). These advances are rooted in key developments in NLP. The transformer architecture revolutionized the field by relying entirely on attention mechanisms, allowing models to capture long-range dependencies and contextual information (Vaswani et al., 2017). Encoder-only models such as BERT (Devlin et al., 2019) further advanced deep contextualized language modeling, while subsequent improvements (Warner et al., 2024) increased efficiency and performance on classification and retrieval tasks. Despite their strengths, encoder-only models are not designed for large-scale semantic similarity tasks. Sentence-transformers addressed this limitation by refining encoder-only models with siamese or triplet architectures, enabling efficient and precise similarity assessments (Reimers and Gurevych, 2019). They produce embeddings that reflect semantic similarity, providing a powerful foundation for neural topic models. Building on these advances, modern topic modeling approaches combine contextual embeddings with clustering techniques. For instance, centroid-based methods group embeddings into clusters and interpret words closest to the centroid as representative of the topic (Sia et al., 2020; Angelov, 2020). While computationally efficient, this assumption can be fragile, since real-world clusters do not always follow spherical distributions, leading to potential misrepresentation of topics (Grootendorst, 2022). A promising approach for topic modeling based on contextual embeddings, addressing centroid-based clustering issues, is BERTopic (Grootendorst, 2022).

#### 2.2.2 BERTopic

BERTopic structures topic modeling into five sequential steps. First, document embeddings are generated using a pre-trained sentence-transformer, leveraging the benefits of advancements in modern language models (Grootendorst, 2022; Gu et al., 2024). Second, dimensionality reduction is applied to improve computational efficiency and clustering accuracy (Allaoui et al., 2020). Third, the reduced embeddings are clustered into semantically similar groups, i.e., topics. Fourth, documents are tokenized. Finally, token importance within topics is determined by assessing class-based tfidf (c-tfidf). c-tfidf weighs the importance of tokens within topics, enabling a more efficient extraction of topic representations.

Despite its advantages, BERTopic also faces challenges. It tends to produce a multitude of closely interconnected topics which may vary upon repeated modeling attempts (Egger and Yu, 2022). This variability contributes to inconsistency in producing meaningful results, further complicated by the complexity of interpreting hyperparameters, hindering troubleshooting and diminishing the reliability of results (Abdelrazek et al., 2023). Moreover, BERTopic assumes that each document relates to a single topic, potentially oversimplifying real-world complexity (Grootendorst, 2022). Additionally, sentence-transformer models used for the document embedding step perform optimally with sentences or paragraphs, while longer documents are truncated (Reimers and Gurevych, 2019). Furthermore, high computation times can result from processing large amounts of data Grootendorst, (2022).

Due to its novelty, applications of BERTopic are still in their infancy. In a financial context, Kim et al. (2022) utilized BERTopic on Item 1A from 10-K filings. They examined whether identified topics can enhance the accuracy of ESG rating predictions and quantify each topic's relative contribution to the final rating prediction. In other contexts, BERTopic has been applied in various studies: Sánchez-Franco and Rey-Moreno (2022) analyzed customer reviews, Abuzayed and Al-Khalifa (2021) explored its application with pre-trained Arabic language models, Egger and Yu (2022) evaluated its performance on Twitter data, and Grigore and Pintilie (2023) extended BERTopic to predict individual's responses to a questionnaire based on their social media activity.

### 2.3 Topic modeling of Item 7 and Item 7A

Our research is driven by several motivations regarding the choice of documents and analysis techniques. Item 7 and Item 7A stand out as particularly crucial sections in 10-K reports (Bhattacharya and Mickovic, 2024). The MD&A section (Item 7) provides a narrative that contextualizes the presented numbers. In this section, management offers its individual perspective, which is essential for understanding the company's strategic direction and potential challenges. Additionally, the MD&A section offers the most leeway and flexibility, making it rich with insights and indicative of future performance Cohen et al., (2020). Item 7A focuses on market risks, containing valuable information regarding the company's prospective performance. Analyzing these sections enables extraction of textual information relevant for predicting future firm performance. While Items 7 and 7A are our primary focus, we also analyze Items 1 and 1A, which are widely recognized for their economic relevance (Jamshed et al., 2025; Kim et al., 2022). This allows us to test FinTextSim's generalizability, with results for Items 1 and 1A reported in the Supplementary material. Whereas most prior work focuses on social media data (e.g., Song et al., 2025; Zheng et al., 2025; Ji and Han, 2022; Deveikyte et al., 2022), we extract and structure firm- and management-specific information from 10-K reports. To operationalize this analysis, we rely on topic modeling (Ranta et al., 2022; Abdelrazek et al., 2023).

Despite methodological advances, applications of topic modeling in finance remain scarce. LDA still dominates applied topic modeling, although more powerful approaches such as BERTopic are available (Egger and Yu, 2021; Blair et al., 2020). To bridge this gap, we benchmark classical models alongside contemporary ones, focusing on BERTopic. We demonstrate that FinTextSim, a finetuned sentence-transformer, substantially enhances BERTopic's ability to produce precise and coherent financial topics. Beyond improving research quality, FinTextSim contributes to the democratization of knowledge-intensive, expert-driven tasks (Zhang et al., 2026; Garćıa-Méndez et al., 2024), enabling more efficient and effective interpretation of disclosures for both analysts and non-experts. It also lays the foundation for aspect-based managerial sentiment analysis, further improving predictive models in valuation and stock price forecasting (Garćıa-Méndez et al., 2023; Ueda et al., 2024).

### 2.4 Corporate performance prediction

Forecasting corporate performance is a central objective in accounting and finance research due to its proven relationship with excess investment returns and capital market efficiency (Ou and Penman, 1989; Cao and You, 2024; Veganzones and Severin, 2025). Traditional approaches relied on statistical, regression-based models (Ou and Penman, 1989). More recently, ML techniques have gained prominence for their ability to learn complex patterns from large-scale financial data. These models uncover economically meaningful relationships between historical financial variables and future performance, generating significant abnormal returns when used for portfolio formation (Hunt et al., 2019; Uddin et al., 2022; Chen et al., 2022). Collectively, these studies highlight the growing potential of ML-based approaches to extract predictive insights that surpass those of human analysts or traditional benchmarks (Campbell et al., 2024; Van Binsbergen et al., 2023; Aoki et al., 2025).

Despite these advances, notable limitations remain. Most existing applications rely predominantly on structured numerical data. While ML models based on financial indicators can correct analyst biases and uncover hidden dependencies (Campbell et al., 2024; Van Binsbergen et al., 2023), they fail to capture forward-looking managerial information that is explicitly communicated through narrative sections such as the MD&A (Aoki et al., 2025). Recent studies have begun to incorporate textual disclosures using ML models to predict corporate performance (e.g., Frankel et al., 2022; Siano, 2025). However, these approaches largely adopt end-to-end predictive frameworks and do not systematically compare alternative textual representations. Although prior work highlights the potential of NLP to improve corporate performance predictions (Peng, 2025; Xinyue et al., 2020; Jun et al., 2022; Theodorakopoulos et al., 2025), evidence on which types of textual representations, particularly topic-based representations, provide incremental value beyond standard financial indicators remains limited.

Our study addresses this gap by integrating financial indicators with topic modeling outputs to assess the incremental informational value of textual representations for corporate performance prediction. Specifically, we integrate topic-document distributions derived from Item 7 and Item 7A of 10-K filings with fundamental financial indicators in a ML framework to predict firms' Return on Assets (ROA). We demonstrate that topic representations derived from BERTopic in combination with FinTextSim yield the most consistent predictive improvements when integrated with financial indicators, particularly in linear models. While several textual representations provide modest gains in more flexible non-linear models, FinTextSim is the only approach that improves performance reliably across both linear and non-linear settings. This finding suggests that domain-specific language models can effectively quantify qualitative disclosures, boosting both interpretability and reliability in corporate performance forecasting.

## 3 Materials and methods

In the following subsections, we outline the materials and methods of our study. This section is divided into several parts: sourcing the dataset, creating an enhanced financial keyword list, training FinTextSim, creating the topic models, presenting the metrics used to evaluate the performance of the topic models, and the description of the downstream task of predicting corporate performance.

### 3.1 Dataset

Our study focuses exclusively on Item 7 and Item 7A of 10-K reports while avoiding survivorship bias. Given their greater significance, we deliberately choose 10-K over 10-Q reports (Griffin, 2003). We source our data from the Notre Dame Software Repository for Accounting and Finance in text-file format, which underwent a “Stage One Parse” to remove all HTML tags.^1^

To avoid survivorship bias, we include 10-K filings of all companies that have been listed in the S&P 500 index between 2016 and 2023. Using a regular expression-based extractor, we isolate the text from the start of Item 7 to the start of Item 8. We refer to this combination of Item 7 and Item 7A as “documents.” To ensure comparability, documents containing fewer than 250 words are discarded.^2^ Additional outlier documents are removed using z-scores, excluding documents more than two standard deviations from the mean length. Text preprocessing methods are applied to improve model performance and comparability across methods (Siino et al., 2024), including replacing contractions as well as removing URLs and numerical characters.

Table 1 summarizes the number of documents at each preprocessing step.

**Table 1:** Dataset.

**Preprocessing-step**	**# Documents**
Extracted documents	4,754
Outlier documents	629
Remaining documents in database	4,125
Number of sentences	2,178,712

As BERTopic assumes single-topic documents and sentence-transformers and NMF perform best on short inputs (Grootendorst, 2022; Reimers and Gurevych, 2019; Chen et al., 2019), we tokenize each of the remaining 4,125 document into individual sentences. This avoids losing information through truncation and prevents misleading single-topic assumptions for multi-topic MD&A sections. As a result, our dataset contains 2,178,712 sentences.

### 3.2 Keyword list

To train FinTextSim, we build on an established keyword framework for financial text. The foundation is the economic anchorword list for 10-K and 10-Q reports proposed by Li (2010), which covers eleven domains.^3^ Subsequent work by Fengler and Phan (2025) expanded this list by identifying semantically related terms with a Word2Vec model trained on MD&A sections of 10-K filings. Building on this evolution, we further refined this list to contain common performance indicators and operational terms. Moreover, we broadened it with a dedicated topic on Environmental Sustainability, reflecting the growing importance of ESG-related disclosures (Giudici and Wu, 2025; Xie et al., 2025).^4^

### 3.3 FinTextSim

To accurately cluster semantically similar financial text, we introduce FinTextSim. FinTextSim is a sentence-transformer model specifically finetuned to enhance contextual embeddings for the financial domain. Given the financial jargon and its domain-specific nuances, off-the-shelf (OTS), general-purpose sentence-transformers fall short. Existing models tailored for the financial domain are primarily optimized for sentiment analysis (e.g., Araci, 2019; Li et al., 2023; Guo et al., 2024). By finetuning FinTextSim on financial text, we aim to improve the quality of generated topics, enhancing semantic coherence and separation between topics, bridging the gap between general-purpose models and the specific demands of financial text analysis.

We construct a labeled dataset from the corpus described in Section 3.1, using a dictionary-based approach that leverages the keyword list from Section 3.2. To this end, we create a keyword-sentence matrix by iterating over each word in every sentence and matching substrings to keywords. This approach allows recognition of variations such as “logistics” or “logistical” for the keyword “logistic.” Sentences containing two or more keywords from a single topic are labeled accordingly. This procedure ensures topic distinctiveness and provides a reliable ground truth for training, consistent with data-centric perspectives on model quality (Di Gennaro et al., 2024). To prevent overemphasis on repeated phrasings, only unique sentences are retained. Finally, our dataset comprises 113,291 labeled sentences. To avoid data leakage, we train the model using a temporal split. Data from 2016–2021 is used for training while data from 2022–2023 is reserved for testing. Following these steps, we obtain 27,388 test- and 85,903 train-sentences. To assess the robustness of FinTextSim to reduced lexical cues, we conduct an additional evaluation in which 50% of the label-inducing keywords are randomly masked in the test set. Masking is applied only at evaluation time while the trained model remains unchanged, allowing us to examine whether learned representations generalize beyond explicit keyword presence. Results of this masked evaluation are reported in the Supplementary material.

FinTextSim is trained using BatchHardTripletLoss, following methods outlined by Reimers and Gurevych (2019) and Devlin et al. (2019). Unlike standard triplet loss, BatchHardTripletLoss dynamically selects the hardest positive (most dissimilar within the same class) and the hardest negative (most similar from a different class) for each anchor in the batch. This strategy forces the model to learn more discriminative embeddings, leading to faster convergence and improved representation quality (Hermans et al., 2017). As base model, we select ModernBERT, a recent advancement in encoder-only architectures (Warner et al., 2024). We adapt it with a mean pooling and a normalization layer to enhance its performance for sentence similarity tasks (Reimers and Gurevych, 2019). Finally, we train FinTextSim with a batch size of 200 and a margin of five. Following this contrastive learning-based training approach, we aim to improve latent semantic discovery of financial topics (Luo et al., 2024).

We evaluate FinTextSim by comparing its embeddings with those generated by AM, MPNET, and distilroberta-finetuned-financial-news-sentiment-analysis (DR), using intra- and intertopic similarity (see Section 3.5.2). Being the most downloaded models for sentence similarity tasks on the Hugging Face website, AM and MPNET serve as robust baselines. DR is the most prominent model for financial sentiment analysis, acting as domain benchmark. To examine embedding structure, we visualize the learned representations using Uniform Manifold Approximation and Projection (UMAP). Compared to dimensionality reduction alternatives, such as t-SNE or PCA, UMAP better preserves both local and global structure (Allaoui et al., 2020; Angelov, 2020). For UMAP, we employ the following essential hyperparameters:

- Minimum distance: 0, to encourage closely grouped data points, facilitating the formation of clusters representing semantically similar documents.
- Distance metric: Cosine similarity, standard for NLP similarity tasks.
- n_neighbors: 125, prioritizing global structures in our data to identify overarching macrotopics as well as hierarchically lower-ranked microtopics (Angelov, 2020).

We share the labeled dataset alongside FinTextSim's training code in the following Github Repository: https://github.com/JehnenS/FinTextSim.

### 3.4 Model creation

#### 3.4.1 Classical approaches

For the classical topic modeling approaches, we follow widely adopted preprocessing steps: stopword removal, lemmatization and term frequency–inverse document frequency (tf–idf) weighting. We remove stopwords using financial domain-specific lists provided by the Notre Dame Software Repository for Accounting and Finance.^5^ Next, we lemmatize words to reduce vocabulary size. We deliberately choose lemmatization over stemming, as it preserves the interpretability of words better (Maier et al., 2018). To capture multi-word expressions, we construct bigrams and trigrams, combining terms that frequently occur together. We then build a dictionary and corpus representation of the texts and apply tf–idf weighting to emphasize informative words. Finally, we employ LDA and NMF with the number of topics fixed at 12, aligning with the number of domains in our keyword list.

#### 3.4.2 Contemporary approaches

For the contemporary approaches, we generate contextual embeddings using FinTextSim, AM and MPNET. Each embedding model is applied within BERTopic under identical settings, ensuring that embedding choice is the only factor influencing performance. Dimensionality reduction is performed using UMAP, which preserves both global and local structures (Allaoui et al., 2020; Angelov, 2020) and scales effectively to large datasets (Angelov, 2020). We configure UMAP with the same settings as in Section 3.3. To strike a balance between clustering efficiency and information retention, we reduce the dimensionality to ten components. For clustering, we adopt Hierarchical Density-Based Spatial Clustering of Applications with Noise (HDBSCAN). HDBSCAN accommodates clusters of varying size and shape, models noise as outliers and avoids forcing unrelated documents into topics (McInnes and Healy, 2017). We use the following hyperparameters:

- Minimum cluster size: 5,000, to prioritize global over highly local topics.
- Minimum number of samples: 50, to reduce the number of outliers by requiring denser cluster formation.

We then vectorize documents using a CountVectorizer, removing financial stopwords. To extract relevant financial topics, we apply c-tfidf weighting, reduce overly common words and incorporate seed words from our keyword list with a weighting multiplier of 50. This guides the model toward generating finance-specific, domain-relevant topics while limiting generic clusters.

### 3.5 Topic model evaluation

To compare the performance of the topic models, we focus on two fundamental tasks (Blei et al., 2003; Song et al., 2025):

1. Topic Quality: Ability to uncover interpretable topics in financial texts.
2. Organizing Power: Organizing and structuring documents into distinct, meaningful groups.

The following subsections detail how we operationalize these tasks and how we adapt evaluation to the financial domain.

#### 3.5.1 Topic quality

To assess topic quality, we use NPMI coherence (Rashid et al., 2019; Yadavilli et al., 2024; Tang et al., 2025; Sun et al., 2026). NPMI measures the strength of association between words by comparing observed co-occurrence with expected independence. Following Röder et al. (2015), NPMI coherence is computed with a sliding window. For classical models, we maintain the default window size of ten. Due to the shorter sentence lengths resulting from stopword removal in classical models, we adjust the window size for BERTopic. Based on the ratio between sentence lengths of BERTopic versus classical models, we set the window size for BERTopic to 20, guaranteeing comparable context coverage. Moreover, we lemmatize BERTopic's input texts and topic representations to reduce the impact of divergent vocabulary sizes. For each model, we use the five most representative words per topic, balancing informativeness with interpretability (Agrawal et al., 2018).

Raw coherence scores alone do not guarantee financial relevance. To address this, we complement them with topic accuracy, evaluated by human experts. For each topic, ten representative sentences are manually annotated to determine whether the topic assignment is correct. Topic accuracy is then defined as the proportion of correctly classified sentences. This approach captures the ability of each model to identify economically meaningful financial topics and generalize to unseen text. In addition, we perform a qualitative analysis of topic assignments to examine strengths and weaknesses of each model in capturing domain-specific semantics.

#### 3.5.2 Organizing power

To assess document organization and clustering performance, we measure intratopic similarity (cohesion within topics) and intertopic similarity (separation across topics). High intratopic similarity combined with low intertopic similarity indicates semantically well-structured and diverse topics.

For classical models, similarities are derived from document–topic distributions. First, documents are assigned to their dominant topic. Next, topic embeddings are computed as means of assigned documents. Intertopic similarity is defined as the cosine similarity between topic embeddings. Intratopic similarity is based on the cosine similarity between each document assigned to the topic and the corresponding topic embedding.

For contemporary models, similarities are computed directly from sentence embeddings. Topic embeddings are calculated as the mean of sentence embeddings per topic. Intertopic similarity reflects pairwise cosine similarities between topic embeddings. Intratopic similarity is defined as the average cosine similarity of sentence embeddings to their topic embedding.

Although similarity scores are computed in different latent representation spaces, all evaluated methods rely on cosine similarity, which is bounded and defined relative to a well-specified neutral reference: vector orthogonality. In both classical topic-distribution spaces and neural embedding spaces, orthogonal vectors correspond to the absence of semantic association. Importantly, our evaluation does not compare absolute cosine similarity magnitudes across model architectures. Instead, we assess relative topic structure within each model, focusing on intratopic cohesion and intertopic separation. These quantities are defined with respect to the model-specific similarity distribution and therefore remain interpretable despite differences in representation geometry. To further mitigate architectural effects, all reported similarity statistics are interpreted relative to their empirical within-model distributions rather than as absolute semantic similarity scores. By evaluating the contrast between intratopic and intertopic similarities, rather than their raw levels, we obtain a scale-independent measure of topic organization. This framing enables meaningful comparison of topic separability across architectures while respecting the distinct geometric properties of their underlying latent spaces.

### 3.6 Downstream task: predictive validity

To assess the predictive value of textual information derived from topic modeling, we conduct a downstream task, evaluating whether the inclusion of topic-document distributions improves company performance prediction. Specifically, we examine the extent to which topics extracted from Item 7 and Item 7A contribute incremental predictive information for future firm profitability.

We define the prediction target as the normalized change in ROA. Following Chen et al. (2022), we normalize by subtracting the average change in ROA over the past four years from the current ROA change. In line with recent literature on corporate performance prediction, we frame the task as a binary classification problem that predicts the direction of ROA change (Peng, 2025). This setup further helps in mitigating heteroscedasticity and outlier sensitivity (Freeman et al., 1982; Ou and Penman, 1989). Consistent with Ou and Penman (1989) and Chen et al. (2022), we exclude observations with model probabilities between 0.4 and 0.6 to remove statistically ambiguous cases and strengthen the predictive signal (Jones et al., 2023; Jun et al., 2022).

The independent variables comprise two components: (1) financial control variables and (2) textual topic features. The financial control variables are based on Swade et al. (2023) and Koval et al. (2024). They comprise 15 features that capture value, growth, profitability, momentum, and size. Focusing on this limited set of features allows us to represent key firm characteristics while preserving the interpretability and visibility of the added textual components. The textual variables are derived from topic-document distributions generated by each topic modeling approach. For classical models, we use the model-implied topic–document distributions directly. For BERTopic, which does not natively provide document-level topic probabilities, we employ HDBSCAN-based approximations of topic distributions. In all cases, document-level topic representations are obtained by averaging sentence-level topic probabilities, yielding vectors that reflect the relative importance of each topic within a document.

We evaluate two predictive models widely used in financial prediction: LR, and XGBoost (XGB). LR serves as a linear benchmark, offering simplicity and interpretability (Gangwani and Zhu, 2024; Żbikowski and Antosiuk, 2021). XGB represents a more sophisticated tree-based model, known for its robustness and performance in financial prediction tasks. Tree-based models offer several advantages, as they are capable of handling high-dimensional data and capturing complex, non-linear interactions among features Levy and O'Malley, (2020); Ho, (1995); Varian, (2014); Geertsema and Lu, (2023). Both ML models are trained using a temporal split. We use data from 2016–2021 for training and data from 2022–2023 for testing. For LR, we perform several preprocessing steps to ensure robust model performance, including removing columns or rows with excessive placeholder or zero values, replacing outlier values, and scaling of features. All preprocessing steps are applied while preventing data leakage and look-ahead bias (Żbikowski and Antosiuk, 2021). As tree-based models can internally manage missing values and are resilient to outliers, we do not apply any form of winsorizing or feature scaling for XGB (Ranta and Ylinen, 2024; Geertsema and Lu, 2023). The final dataset contains 3,454 firm-year observations, with 2,568 for training and 886 for testing. We apply balanced class weighting to mitigate minor class imbalance (43.3% positive, 56.7% negative), which is consistent across training and test set.

We evaluate predictive performance using Accuracy, F1-score, and the Area Under the Receiver Operating Characteristic Curve (ROC-AUC). Following Chen et al. (2022) and Carpenter and Bithell (2000), we assess statistical significance of ROC-AUC differences by constructing bootstrap *p*-values for deviations from 50%, i.e. a random guess. Specifically, we generate 10,000 bootstrap samples of equal size to the original test set. The *p-*value is defined as the proportion of the bootstrap AUCs that are below 50%.

For each ML model, we compare seven different inputs: a baseline model that relies solely on financial variables and six text-enhanced models that integrate topic-document distributions from distinct topic modeling approaches. This design enables a direct comparison of the incremental predictive power of textual representations, revealing which topic modeling approach most effectively contributes to corporate performance prediction. Additionally, by applying both linear and non-linear classifiers, we can assess how the benefit of textual features interacts with model complexity.

## 4 Results and discussion

We structure the results and discussion section according to our research questions:

RQ1 FinTextSim: Leveraging the quality of contextual embeddings for the financial domain.
RQ2 Topic Quality: Creating qualitative, coherent topic representations.
RQ3 Organizing Power: Organizing large financial textual datasets.
RQ4 Improving corporate performance prediction with textual data.

The results are presented and contextualized in the following subsections.

### 4.1 FinTextSim—Leveraging contextual embeddings for the financial domain

**Figure 1:**
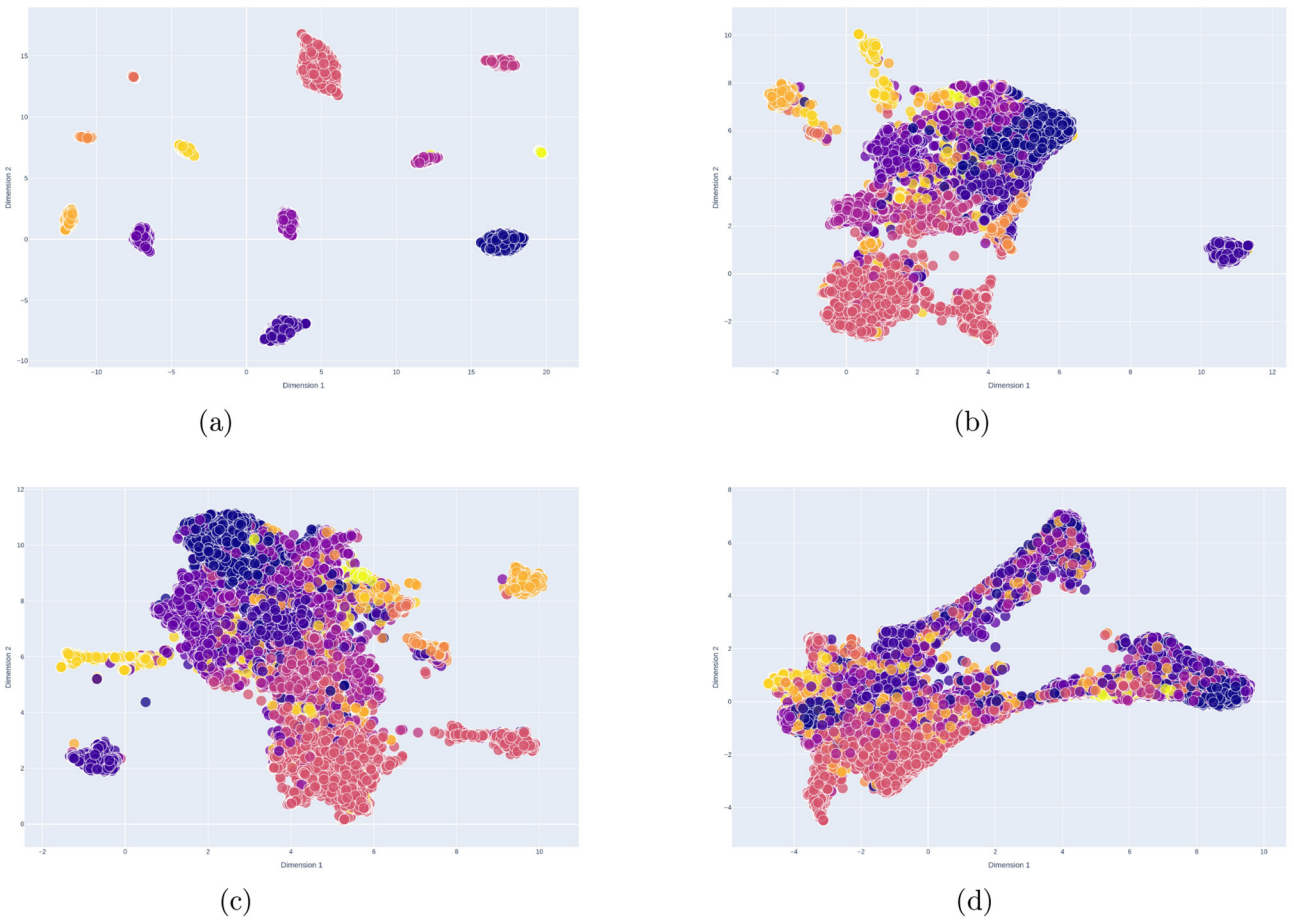
UMAP reduced sentence embeddings **(a)** FinTextSim vs. **(b)** AM vs. **(c)** MPNET vs. **(d)** DR on the test dataset. The colors of the datapoints represent a topic from the keyword list.

FinTextSim generates substantially improved clusters and notably reduces the number of outliers compared to standard embedding models. As illustrated in Figure 1 and Table 2, FinTextSim (Figure 1a) achieves a marked increase in intratopic similarity while simultaneously lowering intertopic similarity relative to AM, MPNET, and DR (Figures 1b–d) on the test dataset.

**Table 2:** FinTextSim vs. OTS embedding models: intra- and intertopic similarity on test dataset.

**Model**	**Intratopic similarity ↑**	**Intertopic similarity ↓**	**Outliers within BERTopic ↓**
FinTextSim	0.998	–0.075	240,823
AM	0.584	0.563	781,965
MPNET	0.614	0.625	784,225
DR	0.773	0.883	1,332,620

Specifically, FinTextSim attains an intratopic similarity of 0.998, substantially exceeding AM (0.584), MPNET (0.614), and DR (0.773). At the same time, FinTextSim reduces intertopic similarity by more than 108% compared to all baselines, achieving a score of –0.075. In contrast, AM and MPNET yield 0.563 and 0.623, respectively, while DR exhibits the highest intertopic similarity at 0.883. Differences across models are further reflected in the number of outliers generated when combined with BERTopic. AM and MPNET generate 781,965 and 784,225 outliers, respectively. DR performs worst, resulting in more than 1.3 million outliers. In contrast, using FinTextSim leads to only 240,823 outliers, representing a reduction of more than 69% relative to all baselines.

These results show that FinTextSim creates significantly enhanced clusters of semantically similar concepts, characterized by high intratopic similarity and low intertopic similarity. AM, MPNET, and DR show limited ability to capture topic-specific nuances, leading to less differentiated embedding spaces (see Figure 1). In parallel, FinTextSim notably reduces the number of outliers, preserving valuable information that standard embedding models discard. Taken together, these findings suggest that OTS sentence-transformers and models finetuned primarily for financial sentiment analysis are less well suited for semantic clustering of financial text. By explicitly modeling domain-specific semantic structure, FinTextSim provides embeddings that better align with financial topical distinctions.

**Figure 2:**
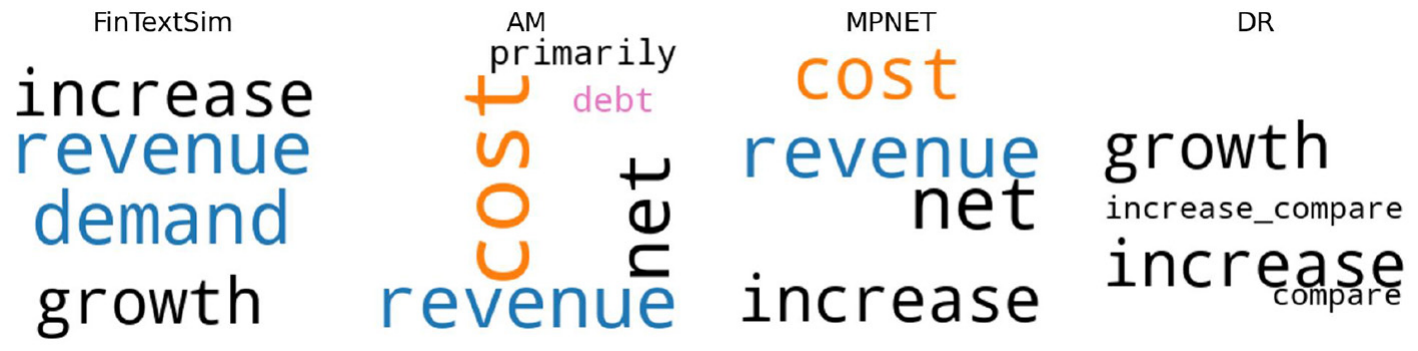
Topic representations for the same sentence using BERTopic with FinTextSim, AM, MPNET, and DR. Words are colored by their assigned topic from the keyword list. Black words are not included in the list. Sentence: “in addition we ended the year with a strong sales backlog up in homes and in dollar value which gives us a strong start for fiscal.”

Turning to a practical example, Figure 2 illustrates topic assignments for the same sentence under BERTopic in combination with FinTextSim, AM, MPNET, and DR. FinTextSim correctly identifies the topic as “Sales,” producing a coherent and interpretable topic representation. In contrast, AM and MPNET assign the sentence to cost- and debt-related topics, reflecting topic confusion and partial concept mixing that limits reliable topic differentiation in this setting. DR assigns the sentence to a diffuse topic lacking clear financial interpretation. This qualitative evidence reinforces the quantitative findings and underscores FinTextSim's advantage in producing interpretable, domain-aligned embeddings that preserve financial topical structure.

### 4.2 Topic quality

As described in Section 3.5.1, we evaluate topic quality using two complementary criteria: NPMI coherence and topic accuracy. While coherence captures statistical word co-occurrence within topics, topic accuracy directly measures whether models correctly identify economically meaningful financial topics. Table 3 reports coherence and topic accuracy for all models.

**Table 3:** Topic quality.

**Model**	**Coherence ↑**	**Topic accuracy ↓**
BERTopic-AM	0.387	0.06
BERTopic-MPNET	0.382	0.23
BERTopic-FinTextSim	0.287	0.81
BERTopic-DR	0.368	0.09
LDA	0.039	0
NMF	0.239	0.11

BERTopic combined with FinTextSim outperforms all alternative approaches in topic accuracy, achieving 81% correct classification across all expert-labeled sentences. In contrast, OTS sentence-transformers achieve markedly lower accuracy. BERTopic with AM achieves a topic accuracy of only 6%, while MPNET reaches 23%. DR exhibits similarly limited performance, achieving 9% accuracy. Classical topic models perform at comparable levels. While NMF reaches 11% accuracy, LDA is unable to correctly identify any topic. A topic-level breakdown shows that FinTextSim consistently identifies most financial topics with high accuracy, whereas baseline models succeed only in narrowly defined, lexically explicit topics such as litigation. This pattern suggests that baseline models rely heavily on surface-level keyword cues, while FinTextSim captures broader domain-specific contextual semantics.^6^ These results highlight that FinTextSim reliably recovers a broad range of economically meaningful financial topics. In comparison, generic embedding models and classical topic models show reduced coverage and consistency, limiting their effectiveness for comprehensive, large-scale financial text analysis.

In terms of raw coherence, BERTopic models outperform classical topic modeling, consistent with Abuzayed and Al-Khalifa (2021) and Egger and Yu (2022). In line with Egger and Yu (2022), O'Callaghan et al. (2015), and Chen et al. (2019), NMF produces more coherent topics than LDA, reflecting its strengths in short-text-modeling (Chen et al., 2019) and handling non-mainstream text (O'Callaghan et al., 2015). LDA, by contrast, generates more general and less domain-specific topics, consistent with O'Callaghan et al. (2015).

During the evaluation of raw coherence scores, an important discrepancy arises: BERTopic with AM, MPNET, and DR achieve higher coherence than with FinTextSim. At first glance, this seems to suggest lower quality for FinTextSim. Yet, this interpretation is incomplete in the financial domain. The paradox arises because coherence does not penalize misclassification, i.e., low topic accuracy. In addition, AM, MPNET, and DR generate a large number of outliers, which simplifies the compression and generation of topics. This artificially inflates coherence while losing valuable financial signals. In contrast, FinTextSim preserves topic distinctions, resulting in fewer outliers and richer topical structures. A further challenge lies in the vocabulary of the financial domain. Key terms often occur as standalone words rather than within a sliding window. Hence, “true” financial topics might suffer from low coherence scores. These factors demonstrate that coherence alone is insufficient to evaluate financial topic models. In line with Grootendorst (2022), who emphasizes that topic evaluation requires both domain expertise and subjective interpretation, we argue that topic accuracy is necessary to capture meaningful financial insights. Standard embedding models within BERTopic and classical topic models exhibit limited ability to correctly identify economically meaningful topics, underscoring their limitations for finance-specific tasks.

**Figure 3:**
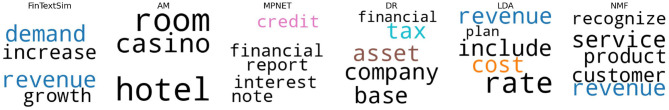
Topic representations - Sales. Original cleaned sentence: “we calculate revpar by dividing hotel room revenue by total number of room nights available to guests for a given period.”

A practical example illustrates this issue. In Figure 3, FinTextSim correctly identifies the topic as “Sales.” AM, MPNET, and DR misclassify the same sentence. Yet, AM receives a coherence score of 0.611, more than double FinTextSim's 0.263. Here, coherence rewards an incorrect classification, undermining interpretability and predictive utility.

**Figure 4:**
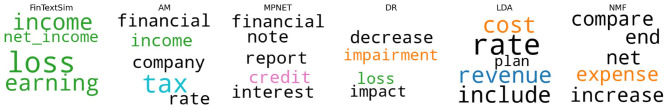
Topic representations - Profit and Loss. Original cleaned sentence: “reported operating profit of million in was million or higher than reported operating profit of million in.”

Figure 4 shows another case: FinTextSim correctly assigns the sentence to “Profit and Loss.” AM associates it with foreign currency and NMF is unable to identify a financial topic at all. Nevertheless, AM (0.528) and NMF (0.341) achieve higher coherence scores than FinTextSim (0.261).

Overall, our findings highlight that domain-specific embeddings are essential for generating high-quality topic representations in financial text applications. Standard coherence metrics systematically undervalue accurate domain-specific topic assignments, while topic accuracy captures meaningful distinctions. By ensuring precise alignment between text and financial topics, FinTextSim provides the interpretability and reliability required for downstream tasks.

### 4.3 Organizing power

To efficiently organize and structure large collections of documents, maximizing intratopic similarity while simultaneously minimizing intertopic similarity is desirable. The results for intra- and intertopic similarity of our models are displayed in Table 4. These metrics are computed within each model's latent space and interpreted relatively, focusing on the contrast between cohesion and separation rather than absolute similarity values.

**Table 4:** Topic similarities.

**Model**	**Intertopic similarity ↓**	**Intratopic similarity ↑**
BERTopic-AM	0.465	0.596
BERTopic-MPNET	0.511	0.656
BERTopic-FinTextSim	–0.034	0.939
BERTopic-DR	0.745	0.948
LDA	1	0
NMF	0.202	0.881

BERTopic combined with FinTextSim consistently achieves the strongest balance between cohesion and separation, producing highly coherent topic clusters (intratopic similarity 0.939) while maintaining strong separation between topics (intertopic similarity –0.034). This demonstrates that FinTextSim captures domain-specific distinctions in financial text, forming distinct and semantically meaningful clusters. By contrast, generic OTS sentence-transformers produce weaker topic structure. Both AM and MPNET exhibit moderate intratopic similarity (0.596 and 0.656) but substantially higher intertopic similarity (0.465 and 0.511), indicating that topics are less well-separated and concepts are partially conflated. DR shows high intratopic similarity (0.948), yet its elevated intertopic similarity (0.745) points to limited topic differentiation. Classical topic models struggle as well. LDA collapses all sentences into a single dominant topic, resulting in maximal intertopic similarity and minimal intratopic similarity. NMF produces higher intratopic similarity than LDA, but intertopic similarity remains at a moderate level, indicating partial topic mixing.

**Figure 5:**
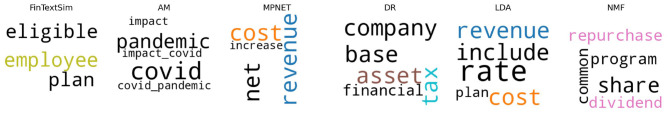
Topic representations - HR. Original cleaned sentence: “in the fourth quarter we recognized our frontline employees for their commitment and contributions to their communities during the pandemic with a award that was paid in January.”

Overall, these results highlight the importance of jointly evaluating intratopic and intertopic similarity. FinTextSim consistently forms clear and well-structured topic clusters, outperforming general-purpose embeddings, domain-specific sentiment-baselines, and classical topic models. Figure 5 illustrates this advantage in practice. FinTextSim correctly identifies the sentence as belonging to the “HR” topic, ensuring a precise and domain-relevant assignment. The alternative models associate the sentence with broader or mixed topics, failing to recover this specific financial concept. Such topic ambiguity manifests in higher intertopic similarity and lower intratopic similarity, underscoring the limitations of OTS sentence-transformers, sentiment-focused financial embeddings, and classical topic models for fine-grained financial semantic clustering.

### 4.4 Predictive validity

As presented in Section 3.6, we evaluate the performance of our corporate performance predicting ML models using accuracy, F1-Score and AUC-ROC. The results are reported in Table 5.

**Table 5:** ML performance comparison across feature sets and models.

**Feature set**	**Accuracy**	**F1 score**	**ROC-AUC**
**LR**			
Financial	***69.2***	57.8	68.8
Financial + AM	63.8	53.3	64.6
Financial + MPNET	66.9	56.5	65.8
Financial + FinTextSim	68.6	**59.9**	***70.8***
Financial + DR	66.5	53.9	66.7
Financial + LDA	67.4	55.6	67.7
Financial + NMF	66.2	56.4	69.0
**XGB**			
Financial	63.6	60.3	67.2
Financial + AM	66.3	***62.6***	67.4
Financial + MPNET	64.8	58.0	66.7
Financial + FinTextSim	66.0	61.2	**68.6**
Financial + DR	65.7	59.4	67.6
Financial + LDA	**67.0**	62.2	67.6
Financial + NMF	66.7	60.8	68.2

Bold values indicate the best performance within each model. Values highlighted in bold and italic indicate the best performing model-feature combination overall. Values are in percent.

For LR, FinTextSim delivers the strongest and most consistent improvements. Topic features derived from BERTopic combined with FinTextSim yield the highest ROC-AUC (70.8) and F1-score (59.9), representing an improvement of approximately two percentage points over the financial baseline. These gains are statistically significant and reflect simultaneous improvements in both precision and recall. In contrast, text features derived from OTS sentence-transformers and models finetuned for financial sentiment analysis reduce predictive performance relative to the financial baseline. Classical topic models offer only marginal or inconsistent improvements. Overall, these results suggest that weak or noisy topic representations do not reliably contribute predictive signal and may adversely affect linear classifiers.

Results under XGB paint a complementary picture. As a more expressive, non-linear model, XGB is better able to accommodate heterogeneous feature quality. Several text-based feature sets yield modest, statistically significant improvements over the financial baseline. Nevertheless, FinTextSim remains the most consistent performer across evaluation metrics, achieving the highest ROC-AUC while maintaining competitive accuracy and F1-score. Importantly, no alternative model matches FinTextSim's joint gains across linear and non-linear classifiers.

Taken together, these findings highlight two key insights. First, predictive gains from textual topic features are highly sensitive to embedding quality, particularly in linear models where noise cannot be absorbed through model complexity. Second, FinTextSim is the only embedding approach that improves predictive performance robustly across both LR and XGB. FinTextSim's superior predictive validity aligns with its stronger intrinsic characteristics, namely higher topic quality and cluster separation. These properties are therefore not merely internal measures of representational quality but translate directly into extrinsic predictive utility. This demonstrates that domain-specific embeddings can effectively extract latent, forward-looking information embedded in corporate narratives. On the other hand, classical, general-purpose or sentiment-focused models tend to provide weaker predictive signals in our setting.

Our results are consistent with and extend previous findings in the earnings and profitability prediction literature. The accuracy and ROC-AUC values reported in our study exceed most previous work, where accuracy typically ranges between 57% and 64% and AUC scores around 68% (Anand et al., 2019; Baranes et al., (2019); Xinyue et al., (2020); Jones et al., (2023); Chen et al., (2022). For example, Jones et al. (2023) report an AUC of 68.4% for LR, while Chen et al. (2022) achieve between 67.5% and 68.7%. Compared to these benchmarks, our FinTextSim-based model demonstrates superior predictive validity using a lightweight LR framework and a wide range of model probabilities, whereas many studies only focus on the first and last quintile (Jones et al., 2023; Jun et al., 2022). This reinforces that the observed improvement is not an artifact of model complexity or sample selection but stems from the added informational value of textual features derived from domain-specific contextual embeddings. Contrary to our expectations, LR outperforms XGB, diverging from prior work Amel-Zadeh et al., (2020); Rossi and Utkus, (2020); Levy and O'Malley, (2020); Zhu et al., (2025). We attribute XGB's comparatively weaker performance to our deliberately parsimonious feature set, which limits the scope for higher-order interactions.

Overall, our findings confirm that textual representations can meaningfully enhance the prediction of corporate performance when generated by a domain-adapted language model. FinTextSim captures subtle linguistic signals reflecting managerial expectations, strategic orientation, and forward-looking disclosures that are otherwise omitted in numerical data. By integrating such qualitative cues into financial prediction tasks, we demonstrate that corporate narratives contain actionable, forward-looking information that can improve the predictive power of conventional forecasting models and contribute to a more holistic understanding of firm performance.

### 4.5 Wrapup of results and discussion

We find that BERTopic is highly effective on financial text when combined with FinTextSim. AM, MPNET, DR, and classical topic models tend to produce broader and less differentiated topics, limiting their ability to capture critical financial aspects and resulting in gaps in topical coverage. Only when paired with FinTextSim, BERTopic produces clear, distinct clusters of financial topics, minimizing misclassifications and enhancing interpretability. Conceptually, this aligns with Das et al. (2017), who observed that financial text represented with expert keywords often exhibits almost linearly separable structures. Furthermore, our results support (Dong et al., 2024; Gu et al., 2024; Wang Y. et al., 2024; Hajek and Munk, 2024), demonstrating that finetuning on a domain-specific dataset improves both model performance and domain-specific understanding. While general-purpose embeddings often exhibit biases and limited coverage of specialized financial terminology (Sun et al., 2026; Hajek and Munk, 2024), models finetuned for financial sentiment analysis also appear less effective for robust topic modeling and semantic clustering. In contrast, domain-adapted models like FinTextSim produce sentence embeddings that better capture topic-specific nuances and context (Wang Y. et al., 2024), emphasizing that relying on alternatives may compromise reliability and introduce systematic errors (Sun et al., 2026). The hyperparameter choices for UMAP and HDBSCAN (see Section 3.4.2) are critical to our results. While we prioritized capturing global structures and macrotopics, these settings succeeded only with FinTextSim, which provided high-quality, pre-separated embeddings for financial text. AM, MPNET, and DR exhibit substantially higher outlier rates and produce less distinguishable topic structures under the same settings. This further highlights a unique advantage of FinTextSim: its domain-adapted representations not only enhance intratopic and intertopic similarity but also enable dimensionality reduction and clustering methods to effectively capture macro-level topic structures, reinforcing its suitability for financial text analysis where both clarity and interpretability are paramount.

Beyond intrinsic topic quality, our results show that improved textual representations translate into tangible predictive benefits. For LR, topic features generated by BERTopic in combination with FinTextSim yield a statistically significant improvement over purely financial features, reflected in a two-percentage-point increase in both ROC-AUC and F1-score. In contrast, OTS sentence-transformers, DR, and classical topic models provide no improvement and, in some cases, even degrade performance, indicating that their latent features introduce noise rather than signal. Results under XGB present a complementary picture. As a non-linear learner, XGB is better able to absorb heterogeneous or partially noisy feature sets, leading to modest improvements for several textual representations. Nevertheless, FinTextSim remains the most consistent performer, achieving the highest ROC-AUC while maintaining competitive accuracy and F1-score. No alternative topic modeling approach delivers comparable gains across both linear and non-linear classifiers. Taken together, these findings bridge intrinsic and extrinsic evaluation. The superior topic quality and cluster separation achieved by FinTextSim are not merely internal quality measures but translate into robust predictive utility, particularly when model capacity cannot compensate for weak representations. Hence, we conclude that semantic differentiation between sentence representations not only contributes positively to topic modeling (Wang Y. et al., 2024), but also to corporate performance prediction. Therefore, we partially support prior literature suggesting that NLP can enhance corporate performance prediction. However, our evidence reveals that such improvements are realized only when domain-specific representations are employed. Together, these findings position FinTextSim as a bridge between qualitative disclosure analysis and quantitative forecasting, highlighting the promise of domain-adapted language models in advancing the methodological frontier of textual analysis in accounting and finance.

Evaluating topic models remains challenging (Zhao et al., 2021). Our analysis reveals the limitations of standard coherence metrics. BERTopic with AM, MPNET, and DR attain higher raw coherence than FinTextSim, yet exhibit low topic accuracy caused by frequent misclassifications. These findings underscore the need for new coherence or topic-quality measures tailored to domain-specific texts.

While BERTopic enhances topic modeling relative to classical approaches, there is still significant room for improvement. The transformer architecture, which BERTopic heavily relies on, may not be fully optimized yet. Thus, more sophisticated and computationally efficient alternatives should be explored (Karami and Ghodsi, 2024). Further advancements in encoder-only models could enhance sentence-transformers by improving their contextual understanding of language (Warner et al., 2024). Moreover, applying domain-specific pre-training methods to optimized BERT variants may deepen the model's understanding of financial language, leading to more effective downstream task performance (Huang et al., 2023). Another promising direction is the integration of topic modeling with generative Large Language Models such as GPT. Although generative models alone do not exhibit competitive performance in topic modeling tasks due to difficulties in handling corpus-level information (Wang R. et al., 2024), hybrid approaches that combine their generalization capabilities with topic modeling frameworks may improve both generalization and textual understanding (Tang et al., 2025).

While our experiments focus on Item 7 and Item 7A of 10-K filings, experiments on Item 1 suggest similar performance, indicating that FinTextSim's effectiveness extends to other sections of 10-K filings.^7^ Considering future improvements for FinTextSim, incorporating diverse high-quality financial sources, such as news, conference call transcripts, and analyst reports could lead to enhanced robustness and adaptability (Mohammed et al., 2025). Additionally, incorporating researcher-labeled data may provide further improvements (Di Gennaro et al., 2024; Tang et al., 2025). These advancements not only improve financial text analysis but also enable topic-specific sentiment extraction, which is highly valuable for performance prediction (Gracewell et al., 2025; Deveikyte et al., 2022; Hajek and Munk, 2024).

In terms of corporate performance prediction, the downstream utility of FinTextSim could be utilized to refine investment strategies, generating excess returns by capturing information beyond raw numerical data. We achieve the best results with a lightweight LR framework and a restricted number of features, highlighting that the predictive gains stem from FinTextSim's improved information quality rather than complex model architecture. Yet, applying more complex models and a richer feature set could further amplify FinTextSim's predictive power and strategic relevance.

## 5 Conclusion

Increased availability of information and enhanced computational capabilities have transformed the analysis of annual reports, recognizing the value embedded within qualitative textual data. Automated review processes, such as topic modeling, are essential for analyzing this data. However, in the financial domain, the use of ML based methods (Ranta et al., 2022), including contextual embeddings, remains underexplored (Senave et al., 2023; Hida and Do Nascimento, 2026). We address these issues by bridging the gap between classical and contemporary topic modeling approaches for Item 7 and Item 7A of 10-K reports from S&P 500 companies in the timeframe between 2016 and 2023. Furthermore, we introduce FinTextSim, a finetuned sentence-transformer enhancing financial text analysis with BERTopic, and demonstrate its value in downstream corporate performance prediction.

Our study reveals the advantages of FinTextSim over OTS sentence-transformer models and demonstrates the benefits of contemporary topic modeling approaches over classical ones. FinTextSim excels at generating distinct clusters of topics, substantially outperforming OTS sentence-transformers and models finetuned for financial sentiment analysis. Additionally, FinTextSim enables BERTopic to identify high-quality, domain-relevant topics, whereas standard embeddings, financial domain baselines and classical topic modeling approaches frequently miss key financial concepts, leading to misclassified documents. Combining BERTopic with FinTextSim further enhances the creation of well-separated clusters of financial topics. This underscores the critical role of domain-adapted embeddings for optimal topic modeling outcomes.

Beyond these intrinsic improvements, we demonstrate that enhanced textual representations also yield tangible benefits for corporate performance prediction. When FinTextSim-derived topic features are incorporated into a LR model predicting the direction of ROA changes, performance improves significantly, achieving a two-percentage-point increase in both ROC-AUC and F1-score over a purely financial baseline. In contrast, features derived from alternative embeddings or classical topic models tend to introduce noise, degrading predictive accuracy. Results under XGB present a more nuanced picture. As a non-linear learner, XGB can partially absorb heterogeneous or noisier textual feature representations, leading to modest improvements for several non-FinTextSim topic modeling approaches. Nevertheless, FinTextSim remains the most consistent performer across both linear and non-linear classifiers, achieving the highest ROC-AUC and stable performance across evaluation metrics. These results establish a direct link between topic quality and predictive validity, confirming that domain-specific textual representations can meaningfully enhance corporate performance forecasting.

Our work offers several key contributions. First, we advance contextual embeddings for the financial domain with FinTextSim, which functions as a domain-adapted information filter, addressing the fundamental information processing and retrieval bottleneck in financial text analysis. By transforming unstructured narratives into structured, semantically rich representations, FinTextSim enhances the quality of extracted information and enables ML models to detect economically meaningful signals often overlooked by human analysts and generic models. Second, FinTextSim strengthens the informational content of textual data, allowing analysts and researchers to derive actionable insights that support efficient resource allocation and more informed decision-making. Third, by bridging classical and contemporary topic modeling techniques, we establish a foundation for methodologically consistent and empirically validated model selection in financial text analysis. Finally, we demonstrate the practical value of FinTextSim in a downstream corporate performance prediction task. Thus, our research lays the foundation for integrating narrative information into valuation and forecasting frameworks, highlighting that qualitative disclosures can complement quantitative financial metrics in predictive applications.

Our study is not without limitations. Direct comparison between classical bag-of-words models and contextual embedding approaches remains challenging due to fundamental architectural differences. Additionally, the evaluation of topic models is inherently complex. Single metrics may be misleading, necessitating a holistic combination of quantitative and qualitative assessment.

Future research should continue refining domain-specific embeddings and topic evaluation metrics. Advancements in transformer architectures, embedding strategies, and hyperparameter optimization may further enhance topic stability and interpretability. Integrating FinTextSim-derived features with richer feature sets and more advanced learning frameworks represents another promising avenue. Ultimately, these developments will strengthen the role of FinTextSim as a semantic information filter, deepening our understanding of how corporate narratives convey actionable, forward-looking economic information.

## Data Availability

Publicly available datasets were analyzed in this study. This data can be found here: https://sraf.nd.edu/data/stage-one-10-x-parse-data/ and Github repository (https://github.com/JehnenS/FinTextSim).

## Footnotes

1 The data can be found at: https://sraf.nd.edu/data/stage-one-10-x-parse-data/.

2 Paragraphs typically consist of 100–200 words. Moreover, sentence-transformers, such as AM and FinTextSim are designed to capture the semantic information of sentences and short paragraphs. Input texts longer than 256-word pieces (approximately 170–210 words) are truncated by default. The 250-word threshold ensures that each document includes at least two paragraphs, enhancing relevance, as shorter texts often lack substantive or complete ideas.

3 The domains encompass sales, cost, profit/loss, operations, liquidity, investment, financing, litigation, employment, tax/regulation, and accounting.

4 The keyword list is presented in a Github Repository under https://github.com/JehnenS/FinTextSim.

5 The stopword lists can be found at: https://sraf.nd.edu/textual-analysis/stopwords/.

6 Detailed topic-level accuracies are reported in the Supplementary material.

7 The results of the experiment are displayed in the Supplementary material.
