# Supplementary material for: FinTextSim: a domain-specific sentence-transformer for extracting predictive latent topics from financial disclosures
Source: Front Artif Intell. 2026 Mar 2;9:1752103. doi: 10.3389/frai.2026.1752103 (PMC12989565; doi:10.3389/frai.2026.1752103)
Supplement: Supplementary file 1 [file Data_Sheet_1.pdf]

Supplementary Material for FinTextSim: A Domain-Specific Sentence-Transformer for Extracting Predictive Latent Topics from Financial Disclosures

TOPIC-LEVEL ACCURACY

Table S1 reports topic-level accuracy for each model, measured as the proportion of correctly classified sentences out of ten expert-labeled sentences per topic.

The results reveal substantial heterogeneity across both topics and modeling approaches. FinTextSim uniquely achieves consistently high accuracy across most financial topics, resulting in an overall topic accuracy of 81%. The only category where FinTextSim does not recover the intended topic is Litigation, which relies heavily on narrow legal terminology rather than broader financial semantics. In contrast, OTS sentence-transformers and the financial domain baseline exhibit highly uneven performance. These models achieve non-zero accuracy primarily for lexically explicit topics such as Litigation and, to a lesser extent, Financing and Tax/Regulation. Outside these cases, accuracy is close to zero, indicating limited ability to generalize across diverse financial topics. Classical topic models show similar weaknesses. NMF identifies Accounting and Cost with moderate accuracy but fails on most other topics, while LDA does not correctly identify any topic in the evaluation set.

Overall, the topic-level results demonstrate that only FinTextSim is capable of recovering a broad spectrum of economically meaningful financial topics. In contrast, baseline embedding models and classical topic models succeed only in isolated, keyword-driven categories. This granular analysis complements the aggregate topic-accuracy results and highlights the importance of domain-adapted representations for reliable financial topic identification.

EXPERIMENT - ROBUSTNESS TO PARTIAL KEYWORD MASKING

This experiment evaluates whether FinTextSim relies primarily on explicit keyword cues or learns contextual semantic representations that generalize beyond surface-level lexical features. As FinTextSim is trained on weakly supervised, dictionary-labeled data, robustness to partial keyword removal constitutes an important validity check. To assess robustness, we construct an alternative version of the test dataset in which 50% of the label-inducing keywords are randomly masked. Masking is applied only at evaluation time while the trained model remains unchanged, ensuring that observed performance differences reflect reliance on contextual semantics rather than direct keyword matching. We evaluate performance using the same clustering-based metrics as in the main analysis, namely intratopic similarity and intertopic similarity.

Table S2 reports results with FinTextSim for the unmasked and masked test sets. Partial keyword masking leads to a moderate decline in both intertopic and intratopic similarity. Crucially, FinTextSim continues to produce coherent and well-separated topic clusters despite substantial removal of explicit lexical cues. This indicates that FinTextSim leverages broader contextual information rather than relying solely on keyword-level supervision. Notably, performance on the masked test set remains substantially stronger than that of all alternative embedding models evaluated on the unmasked test set.

Figure S1 visualizes UMAP projections of sentence embeddings under the masked evaluation setting. Even with partial keyword removal, FinTextSim preserves distinct and interpretable topic clusters, further supporting its ability to generalize beyond keyword-level signals.

Overall, the partial keyword masking experiment provides evidence that FinTextSim captures broader contextual semantics rather than relying on explicit keyword memorization. Although masking label-inducing terms leads to a moderate degradation in clustering quality, the model continues to produce coherent and well-separated topic structures. At the same time, the observed sensitivity to keyword removal is consistent with the nature of expert financial language, which is inherently structured around recurring domain terminology. Instead of indicating shortcut learning, these results align with prior findings that financial text often exhibits near-linear separability driven by systematic, economically meaningful vocabulary usage (Das et al., 2017).

EXPERIMENT ON ITEM 1

To test the generalizability of FinTextSim, we replicate the analysis on Item 1 of 10-K filings. Following the same preprocessing steps outlined in Section 3 of the main paper, the corpus contains 1,380,322 sentences. Training and model evaluation are also consistent with the methods outlined in Section 3 of the main paper.

The only difference is the use of topic-precision instead of topic accuracy. As generic evaluation does not ensure financial relevance and manual annotation of domain experts is both complex and time-consuming, we weight coherence and topic similarities with topic-precision. Topic-precision is calculated using the keyword list from Section 3.2. A topic is considered dominant if it contains at least two keywords from a single financial domain and no more than one from another. This criterion reflects a trade-off between semantic specificity and robustness to noise. By requiring multiple domain-specific indicators, we reduce unjustified matches. Furthermore, we allow limited cross-domain overlap to account for the polysemous nature of words. True positives and false positives are counted accordingly, yielding topic precision:

$$Topic - Precision = \frac{TP}{(TP + FP)} \tag{S1}$$

Topics missing from the keyword list receive a precision of zero. Model-level performance is then averaged across all topics. We incorporate topic precision as follows: NPMI coherence and intratopic similarity are multiplied by topic precision, while intertopic similarity is divided by it and capped at 1. This weighting ensures that structural quality is only rewarded if topics are substantively financial, penalizing models that capture generic or irrelevant clusters.

Topic Quality

Table S3 displays topic-precision, coherence and topic-precision weighted coherence for all models.

BERTopic with FinTextSim achieves the highest topic-precision (0.750), far exceeding all other models. AM and MPNET only partially detect financial topics, while DR, LDA, and NMF capture almost none. This indicates that generic sentence embeddings, sentiment-fine-tuned models, and classical topic models are limited in their ability to extract financially relevant structure from Item 1 disclosures. In contrast, FinTextSim consistently identifies financial topics across multiple Items within 10-K filings, demonstrating strong domain generalization. Raw coherence values appear higher for AM and MPNET. Yet this reflects inflated scores from non-financial topics rather than genuine financial relevance. FinTextSim preserves domain-specific distinctions, which coherence alone cannot capture. When weighted by topic-precision, BERTopic with FinTextSim clearly outperforms all alternatives, underscoring the importance of domain-adapted evaluation.

Organizing Power

Table S4 displays intra- and intertopic similarity.

FinTextSim again dominates, yielding the highest intratopic similarity and lowest intertopic similarity. In contrast, LDA collapses all sentences into one topic, producing degenerate results, while NMF, AM, MPNET, and DR underperform substantially. FinTextSim also reduces the number of outliers compared to AM and MPNET, marking a reduction of more than 61%. These findings reaffirm FinTextSim's ability to generate clear and well-separated topic clusters while preserving valuable financial signals.

Wrapup of Experiment on Item 1

The Item 1 experiment validates the robustness of our main findings. Only BERTopic combined with FinTextSim produces precise, interpretable, and domain-relevant topics. Classical models, models finetuned for financials sentiment analysis, and OTS embeddings miss essential financial signals. In contrast, FinTextSim consistently enhances coverage and structure. This confirms that domain-specific finetuning is key to reliable large-scale financial text analysis.

TABLES

Table S1. Topic-Level Accuracy by Model. Accuracy is measured as the percentage of correctly classified sentences out of 10 expert-labeled sentences per topic.

Topic	FinTextSim	AM	MPNET	DR	LDA	NMF
Sales	90%	0%	0%	0%	0%	0%
Cost	90%	0%	0%	20%	0%	30%
Profit/Loss	80%	0%	0%	0%	0%	0%
Operations	80%	0%	0%	0%	0%	0%
Liquidity	100%	0%	0%	0%	0%	10%
Investment	80%	0%	0%	0%	0%	0%
Financing	80%	0%	80%	10%	0%	20%
Litigation	0%	70%	70%	70%	0%	0%
Employment	90%	0%	0%	0%	0%	0%
Tax/Regulation	100%	0%	70%	10%	0%	0%
Accounting	100%	0%	10%	0%	0%	70%
ESG	80%	0%	50%	0%	0%	0%
Overall Accuracy	81%	6%	23%	9%	0%	11%

Table S2. Keyword Memorization 1.

Dataset	Intertopic Similarity ↓	Intratopic Similarity ↑
Unmasked	0.998	-0.075
Masked (50%)	0.839	-0.045

Table S3. Topic Quality Item 1.

Model	Topic-Precision ↑	Coherence ↑	Weighted Coherence ↑
BERTopic-AM	0.381	0.399	0.152
BERTopic-MPNET	0.405	0.340	0.138
BERTopic-FinTextSim	0.750	0.237	0.178
BERTopic-DR	0.083	0.094	0.008
LDA	0.083	0.023	0.002
NMF	0.083	0.194	0.016

Table S4. Topic Similarities Item 1.

Model	Intertopic Similarity ↓	Intratopic Similarity ↑	Outliers
BERTopic-AM	1 (0.465)	0.230 (0.603)	455,111
BERTopic-MPNET	1 (0.507)	0.266 (0.656)	459,312
BERTopic-FinTextSim	-0.020 (-0.027)	0.692 (0.922)	177,068
BERTopic-DR	1 (0.898)	0.071 (0.847)	0
LDA	1 (1)	0 (0)	0
NMF	1 (0.208)	0.073 (0.880)	0

FIGURES

Keyword Memorization

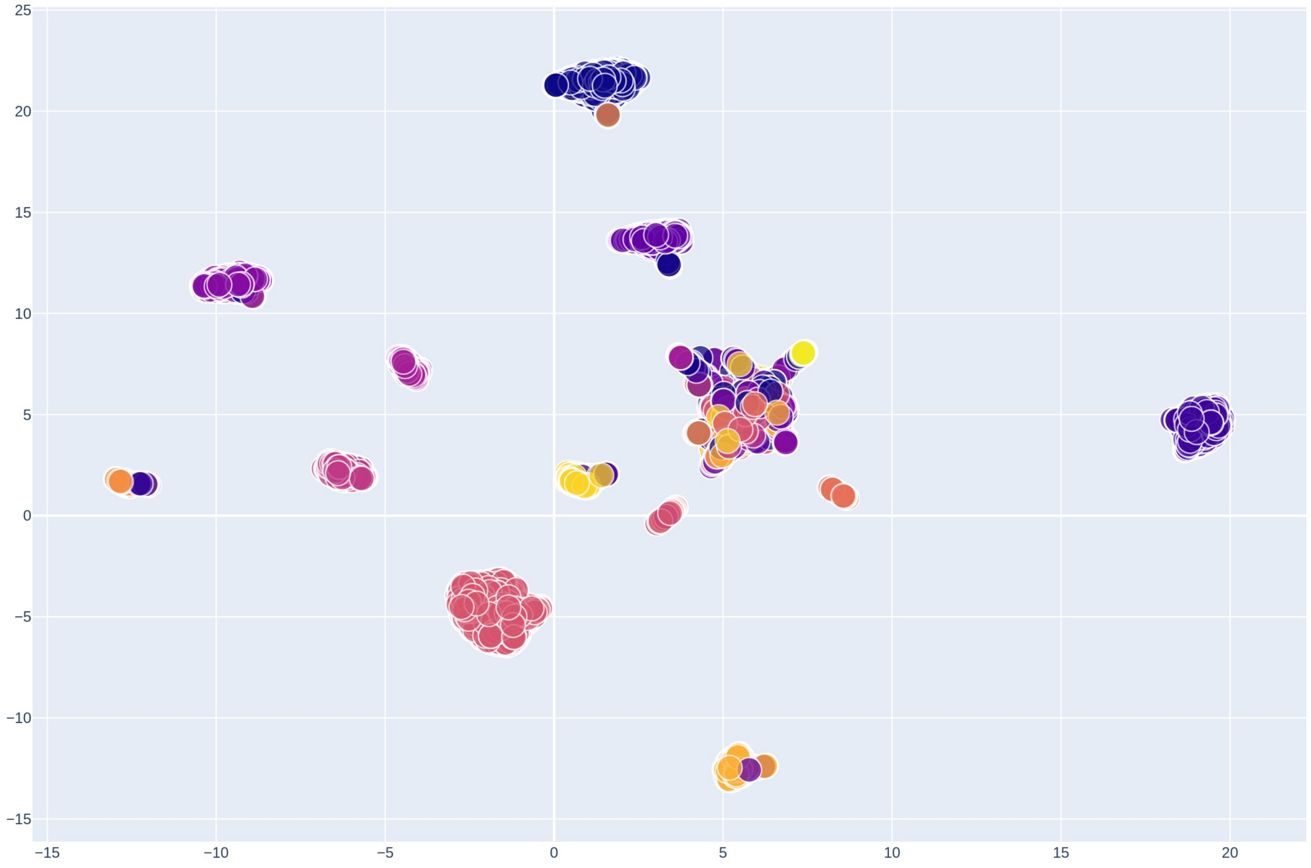

Figure S1: UMAP reduced sentence embeddings FinTextSim on the masked test dataset. The colors of the datapoints represent a topic from the keyword list.

Wordclouds Item 1

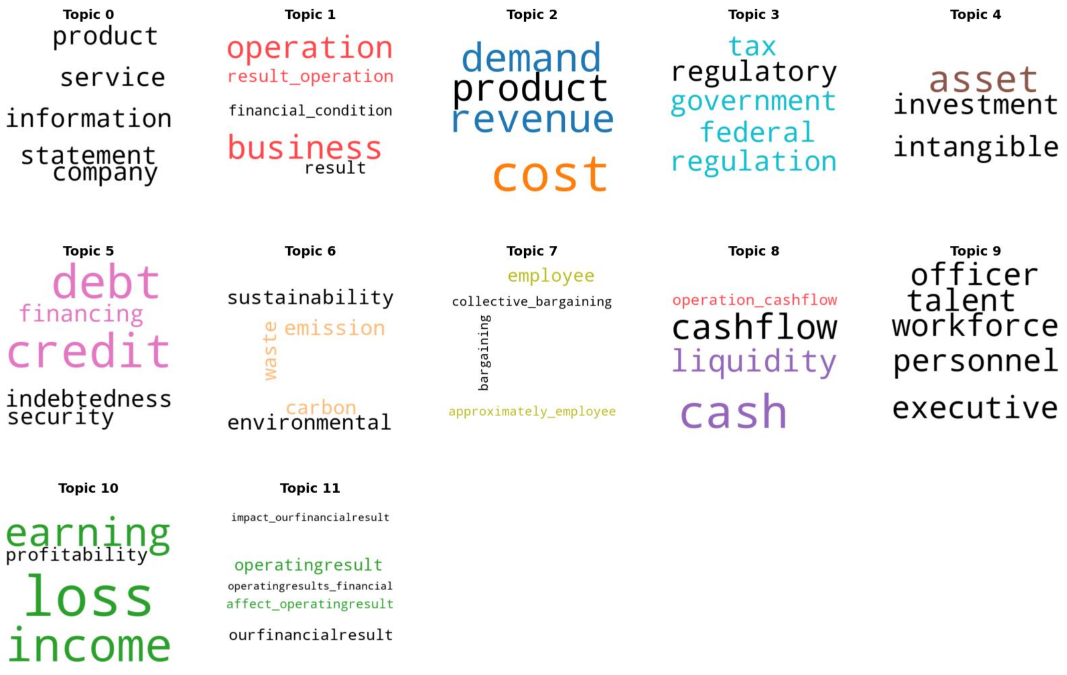

Figure S2: Wordcloud - BERTopic-FinTextSim - Item 1.
The color of each word represents its associated unique topic from the keyword list. Words colored in black are not present in the keyword list. Words colored in darkred are bigrams containing words from multiple keyword domains.

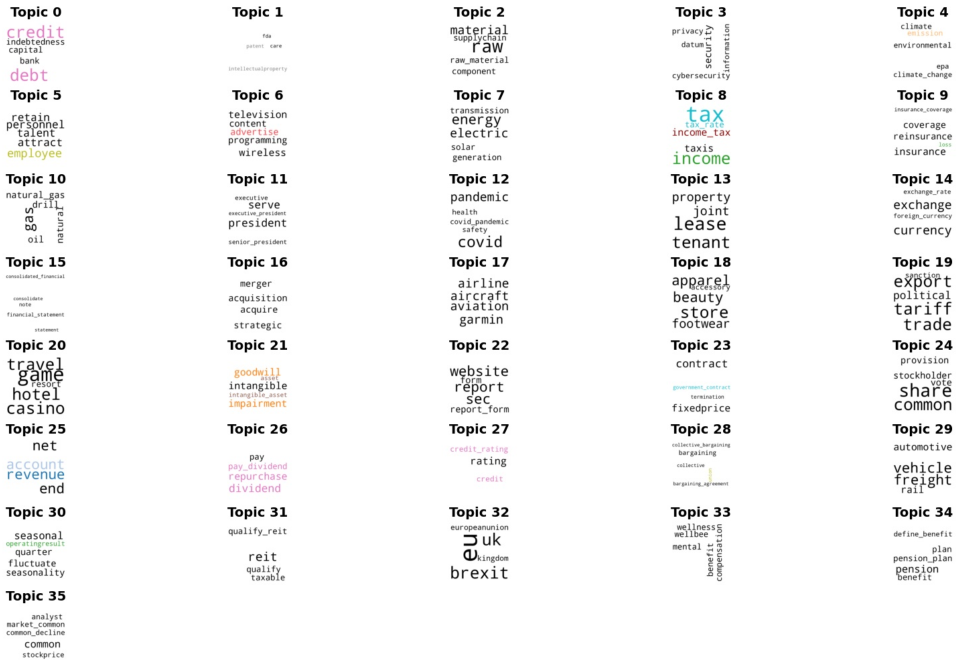

Figure S3: Wordcloud - BERTopic-AM - Item 1.
The color of each word represents its associated unique topic from the keyword list. Words colored in black are not present in the keyword list. Words colored in darkred are bigrams containing words from multiple keyword domains.

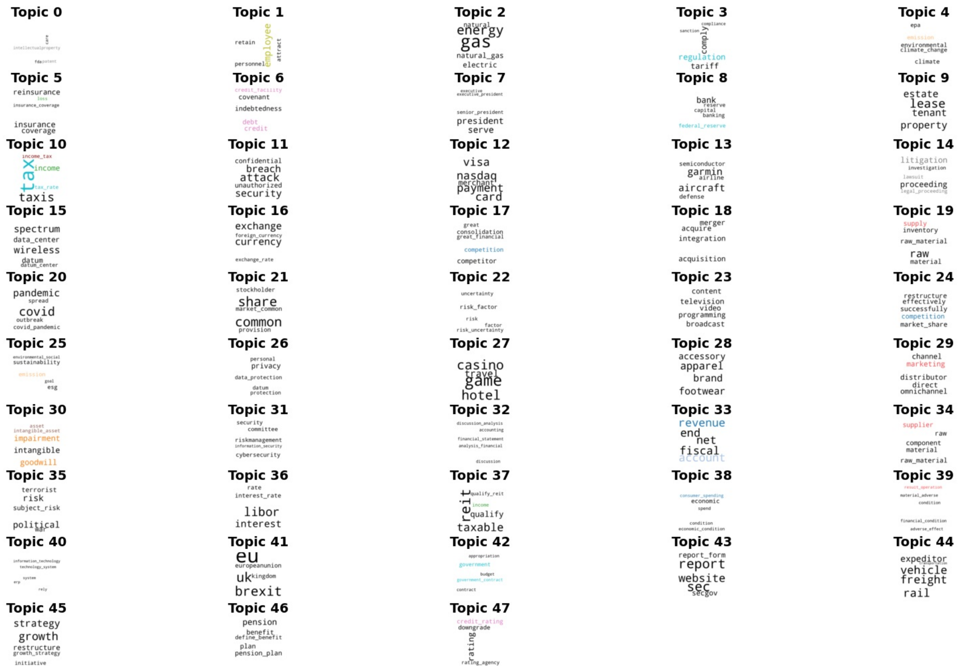

Figure S4: Wordcloud - BERTopic-MPNET - Item 1.
The color of each word represents its associated unique topic from the keyword list. Words colored in black are not present in the keyword list. Words colored in darkred are bigrams containing words from multiple keyword domains.

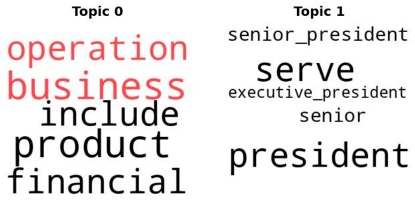

Figure S5: Wordcloud - BERTopic-DR - Item 1.
The color of each word represents its associated unique topic from the keyword list. Words colored in black are not present in the keyword list. Words colored in darkred are bigrams containing words from multiple keyword domains.

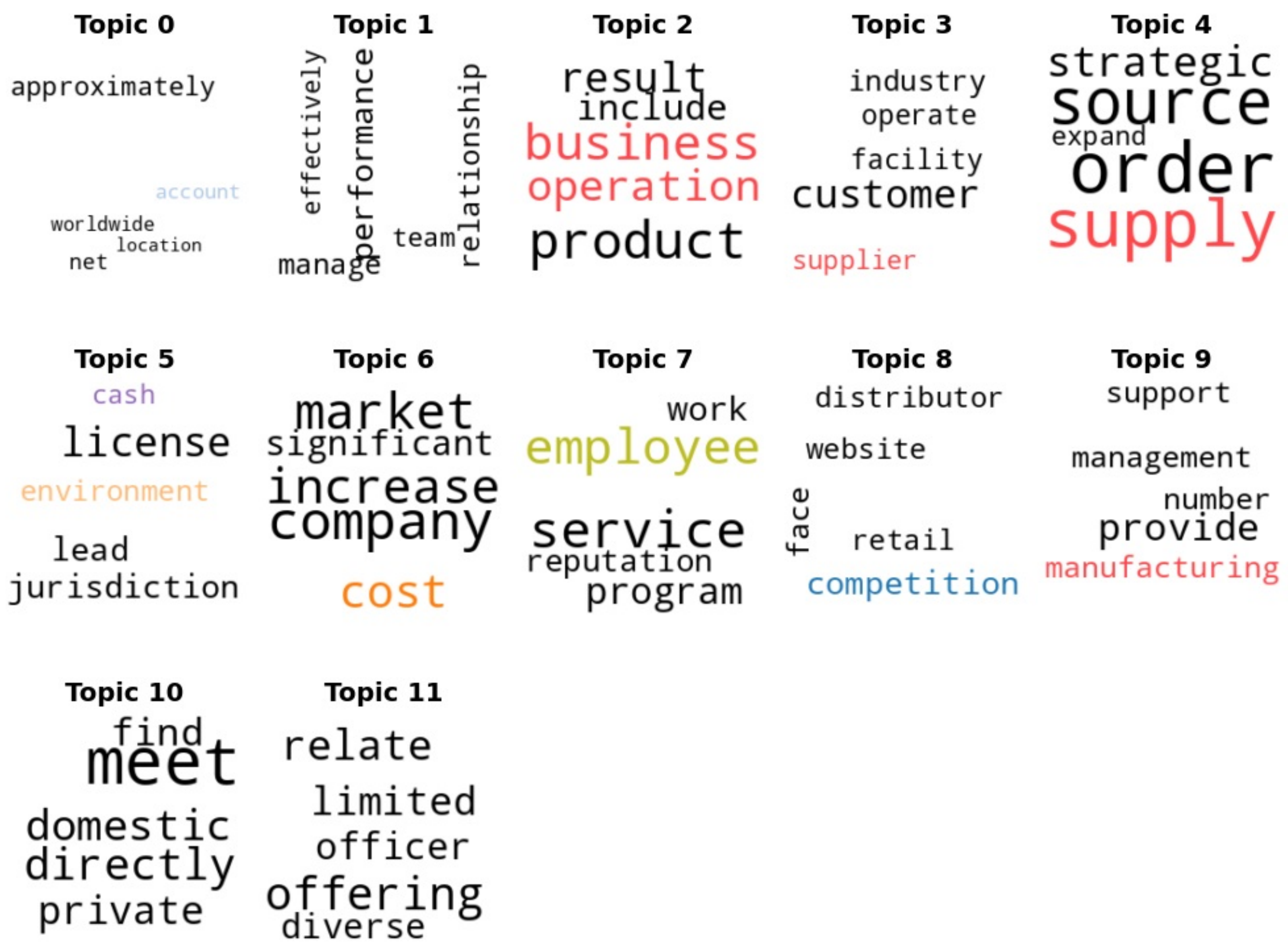

Figure S6: Wordcloud - LDA - Item 1.
The color of each word represents its associated unique topic from the keyword list. Words colored in black are not present in the keyword list. Words colored in darkred are bigrams containing words from multiple keyword domains.

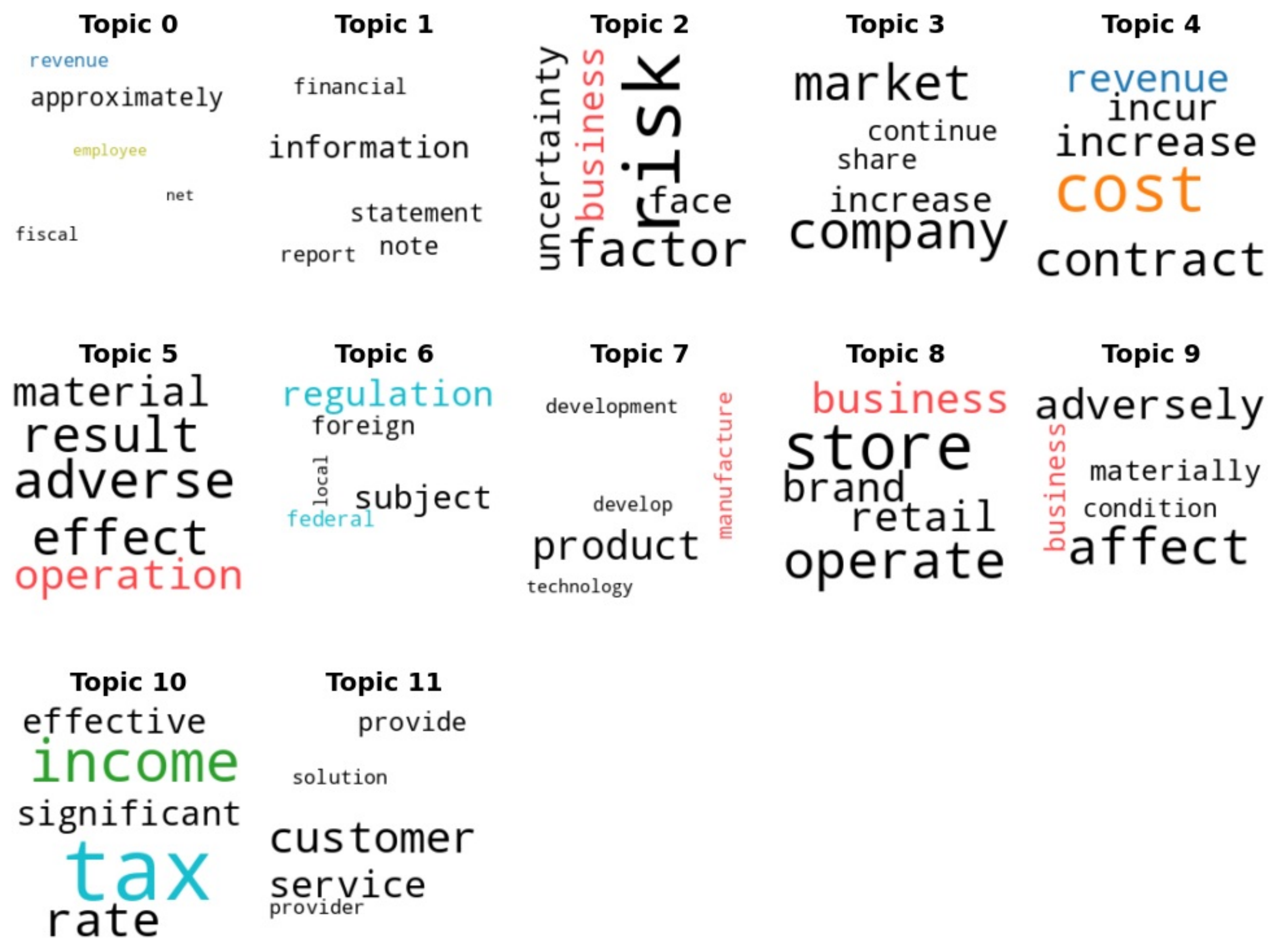

Figure S7: Wordcloud - NMF - Item 1.
The color of each word represents its associated unique topic from the keyword list. Words colored in black are not present in the keyword list. Words colored in darkred are bigrams containing words from multiple keyword domains.

Wordclouds Item 7 and Item 7A

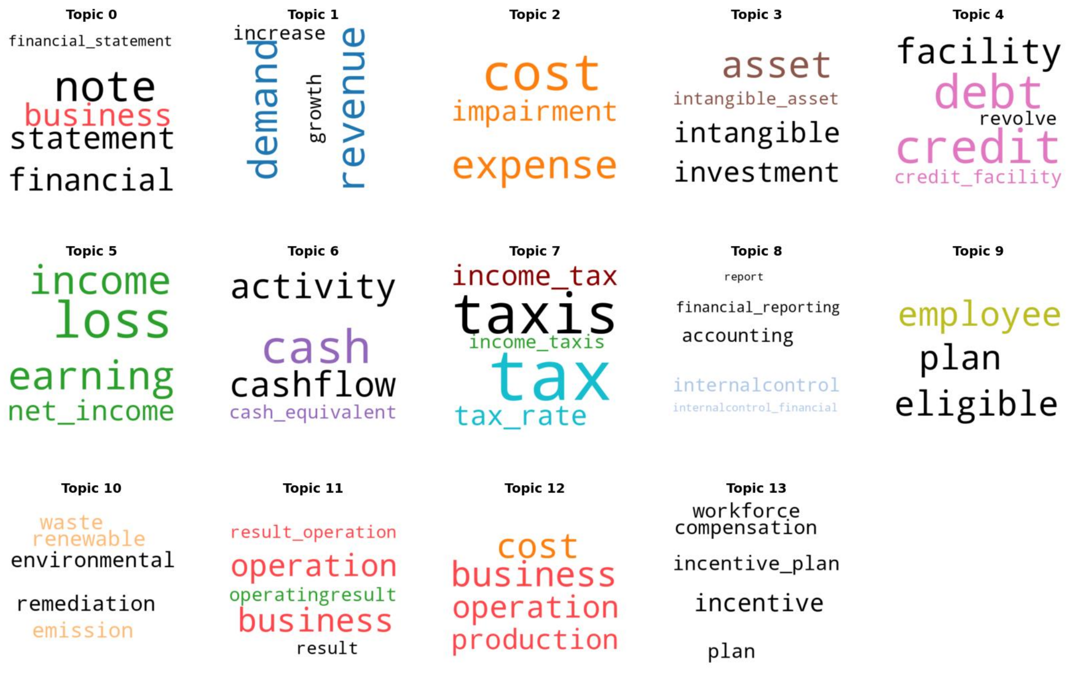

Figure S8: Wordcloud - BERTopic-FinTextSim.
The color of each word represents its associated unique topic from the keyword list. Words colored in black are not present in the keyword list. Words colored in darkred are bigrams containing words from multiple keyword domains.

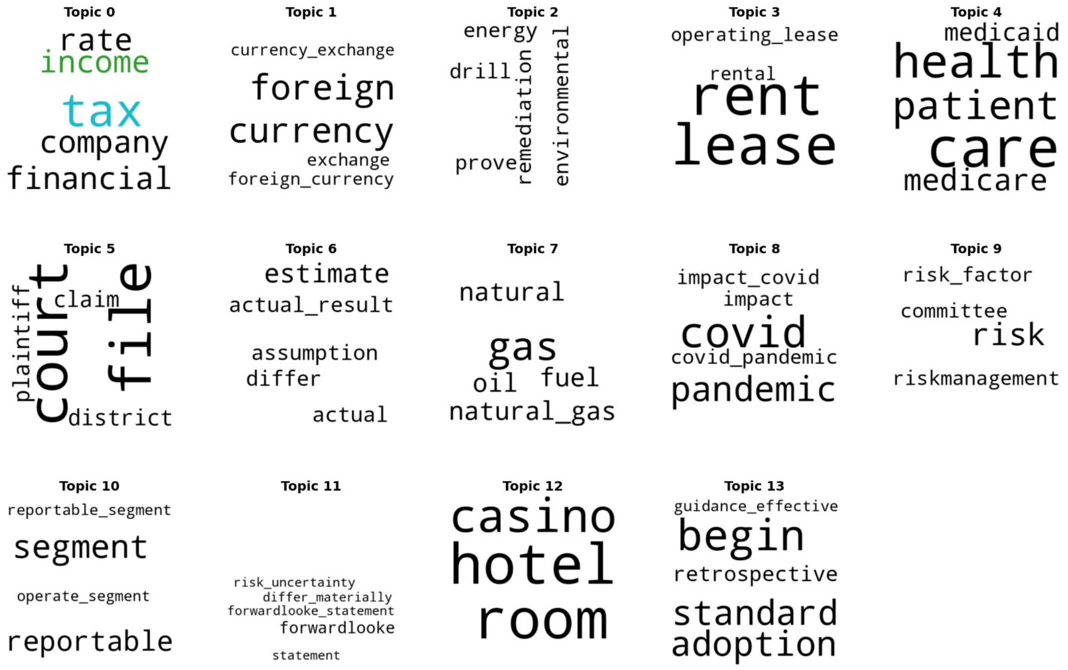

Figure S9: Wordcloud - BERTopic-AM.
The color of each word represents its associated unique topic from the keyword list. Words colored in black are not present in the keyword list. Words colored in darkred are bigrams containing words from multiple keyword domains.

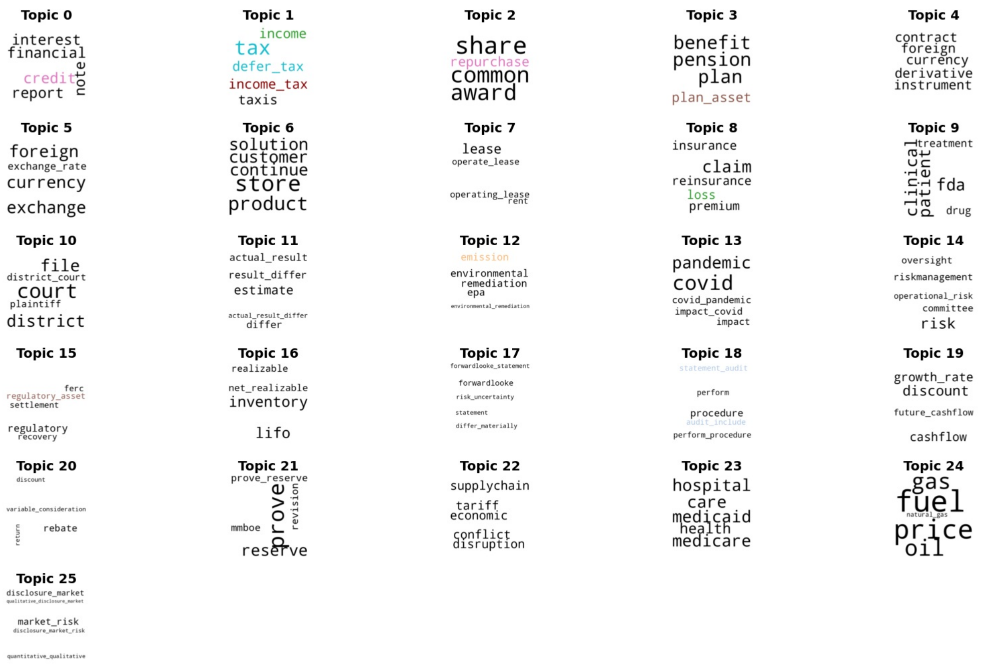

Figure S10: Wordcloud - BERTopic-MPNET.
The color of each word represents its associated unique topic from the keyword list. Words colored in black are not present in the keyword list. Words colored in darkred are bigrams containing words from multiple keyword domains.

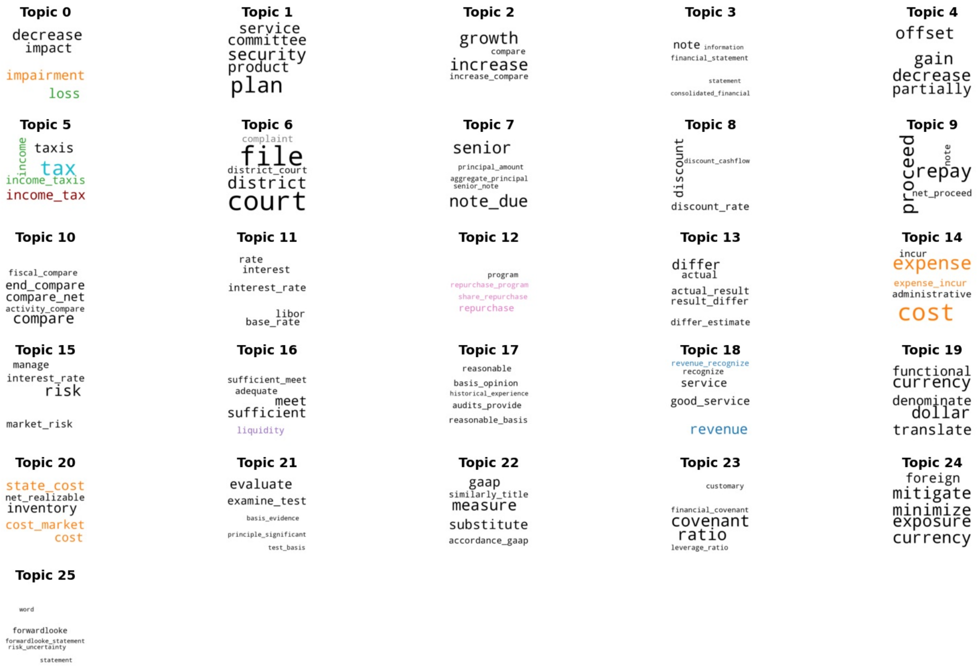

Figure S11: Wordcloud - BERTopic-DR.
The color of each word represents its associated unique topic from the keyword list. Words colored in black are not present in the keyword list. Words colored in darkred are bigrams containing words from multiple keyword domains.

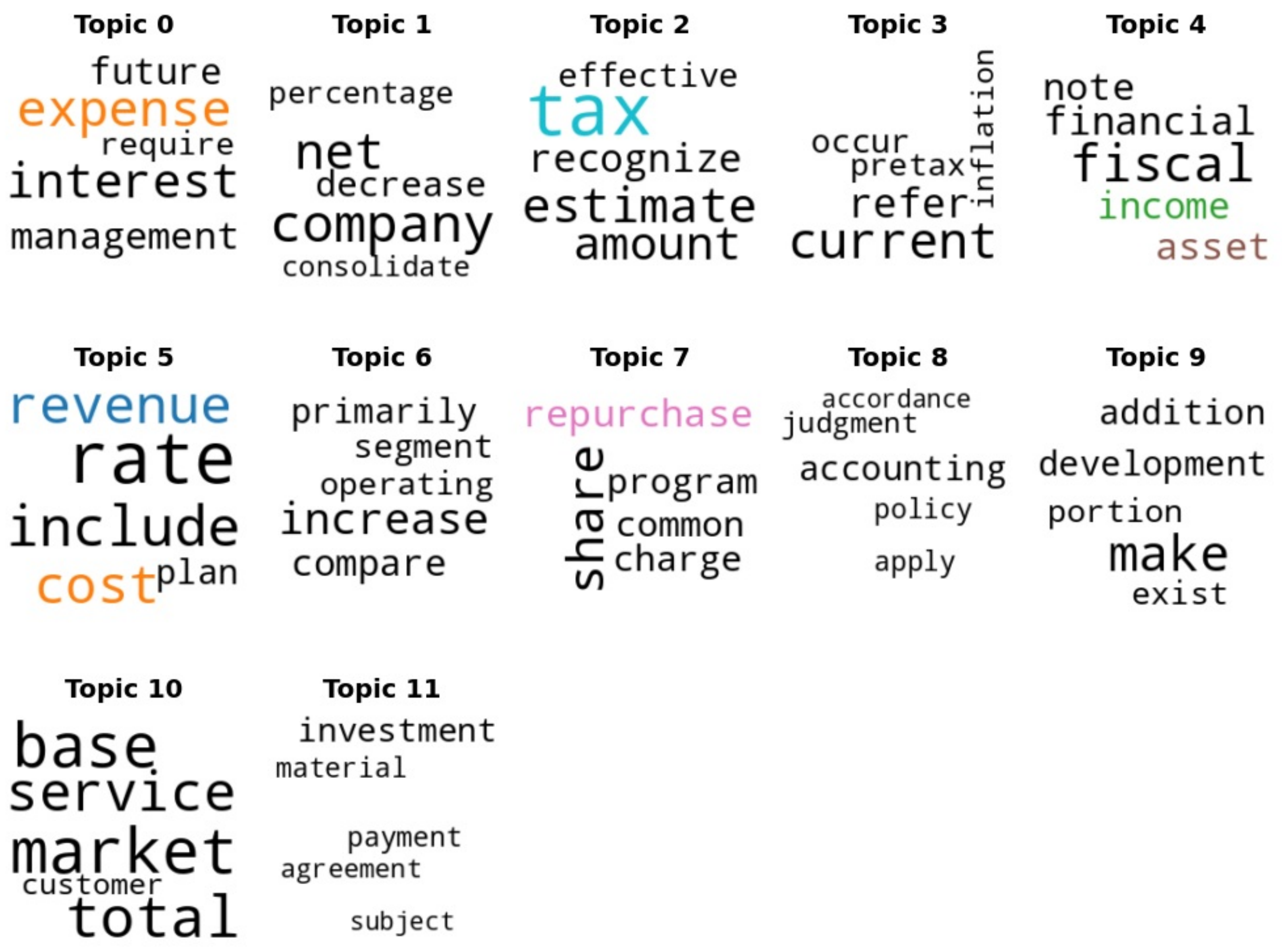

Figure S12: Wordcloud - LDA.
The color of each word represents its associated unique topic from the keyword list. Words colored in black are not present in the keyword list. Words colored in darkred are bigrams containing words from multiple keyword domains.

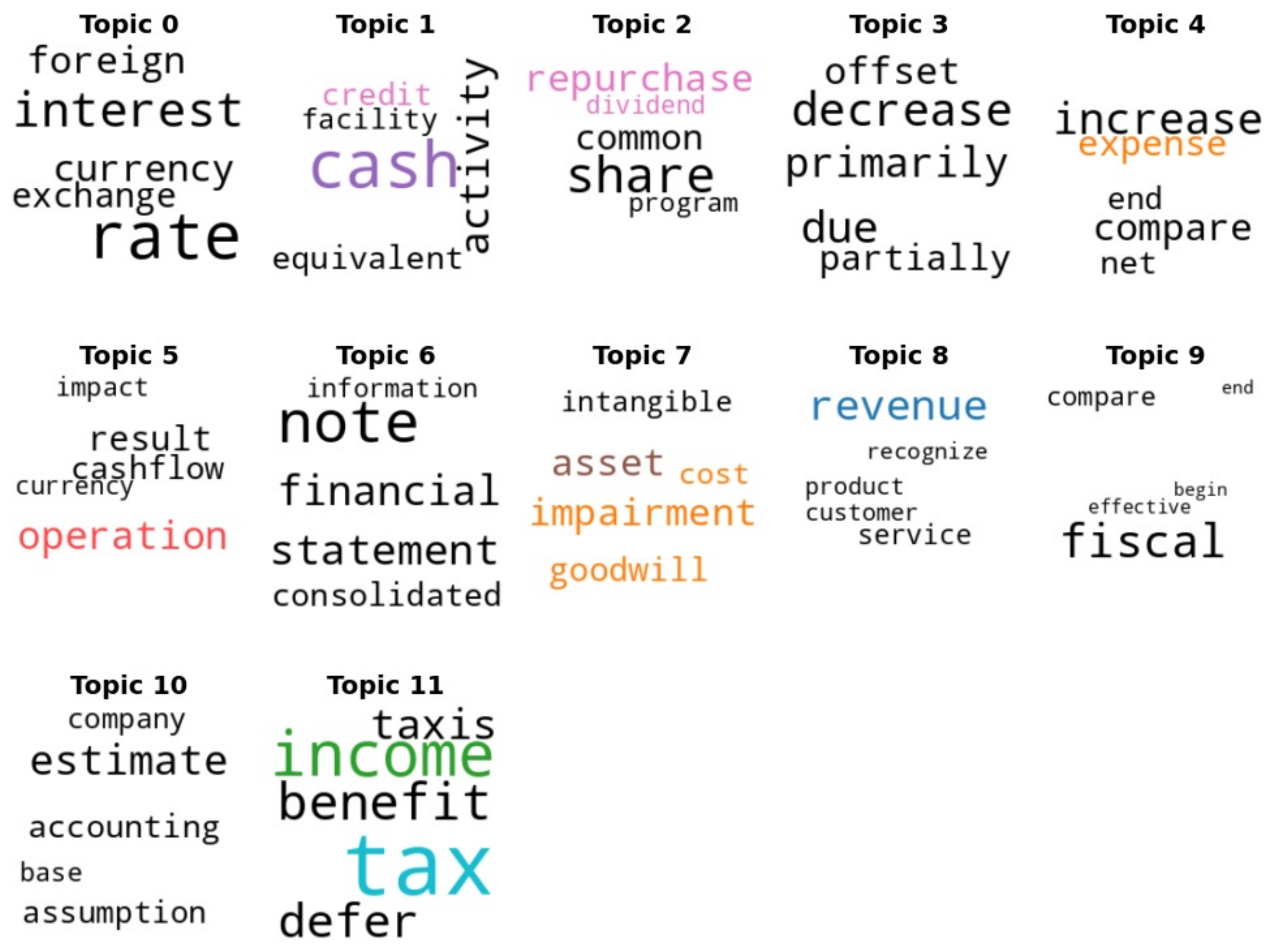

Figure S13: Wordcloud - NMF.
The color of each word represents its associated unique topic from the keyword list. Words colored in black are not present in the keyword list. Words colored in darkred are bigrams containing words from multiple keyword domains.
